# Improved detection of methylation in ancient DNA

**DOI:** 10.1186/s13059-024-03405-5

**Published:** 2024-10-10

**Authors:** Susanna Sawyer, Pere Gelabert, Benjamin Yakir, Alejandro Llanos-Lizcano, Alessandra Sperduti, Luca Bondioli, Olivia Cheronet, Christine Neugebauer-Maresch, Maria Teschler-Nicola, Mario Novak, Ildikó Pap, Ildikó Szikossy, Tamás Hajdu, Vyacheslav Moiseyev, Andrey Gromov, Gunita Zariņa, Eran Meshorer, Liran Carmel, Ron Pinhasi

**Affiliations:** 1 Department of Evolutionary Anthropology, University of Vienna, Vienna, Austria; 2 Human Evolution and Archaeological Sciences, University of Vienna, Vienna, Austria; 3 Department of Statistics, The Faculty of Social Science, The Hebrew University Mount Scopus, Jerusalem, Israel; 4 Facultad de Química y Farmacia, Universidad del Atlántico, Barranquilla, Colombia; 5 Museo Delle Civiltà, Servizio Di Bioarcheologia, Rome, Italy; 6 Dipartimento di Asia, Africa e Mediterraneo, Università degli Studi di Napoli “L’Orientale”, Naples, Italy; 7 Università Di Padova, Dipartimento Dei Beni Culturali, Padua, Italy; 8 Austrian Archaeological Institute, Austrian Academy of Sciences, Vienna, Austria; 9 Institute of Prehistory and Early History, University of Vienna, Vienna, Austria; 10 Department of Anthropology, Natural History Museum Vienna, Vienna, Austria; 11 Centre for Applied Bioanthropology, Institute for Anthropological Research, Zagreb, Croatia; 12 Department of Archaeology and Heritage, Faculty of Humanities, University of Primorska, Koper, Slovenia; 13 Department of Biological Anthropology, Institute of Biology, University of Szeged, Szeged, Hungary; 14 Department of Anthropology, Hungarian Natural History Museum, Budapest, Hungary; 15 Department of Biological Anthropology, Eötvös Loránd University, Budapest, Hungary; 16 Budapest, Hungary; 17 Peter the Great Museum of Anthropology and Ethnography (Kunstkamera), RAS, Saint Petersburg, Russia; 18 Institute of Latvian History, University of Latvia, Riga, Latvia; 19 The Edmond and Lily Center for Brain Sciences (ELSC), The Hebrew University of Jerusalem, Jerusalem, Israel; 20 Department of Genetics, The Alexander Silberman Institute of Life Sciences, The Hebrew University of Jerusalem, Jerusalem, Israel

**Keywords:** Paleogenomics, Methylation, Ancient DNA, Bisulfite treatment, Enzymatic methylation treatment

## Abstract

Reconstructing premortem DNA methylation levels in ancient DNA has led to breakthrough studies such as the prediction of anatomical features of the Denisovan. These studies rely on computationally inferring methylation levels from damage signals in naturally deaminated cytosines, which requires expensive high-coverage genomes. Here, we test two methods for direct methylation measurement developed for modern DNA based on either bisulfite or enzymatic methylation treatments. Bisulfite treatment shows the least reduction in DNA yields as well as the least biases during methylation conversion, demonstrating that this method can be successfully applied to ancient DNA.

**Supplementary Information:**

The online version contains supplementary material available at 10.1186/s13059-024-03405-5.

## Background

Since the sequencing of the first human genome, the field of genetics and genomics has made great strides in our understanding of ourselves from medical breakthroughs to a deeper resolution of our history and evolution. As our grasp of the complex relationship between genetics and gene expression has deepened, the study of epigenetics has become integral to understanding genomics. Epigenetics encompasses the study of regulation of genes that are controlled by factors that do not change the genetic code directly. This includes histone modifications, non-coding RNAs, and modifications of DNA bases. In humans, methylation occurs mostly at one of the four bases, cytosine (C); however, methylation of adenine (A), more common in prokaryotes, has also been suggested to occur in multicellular eukaryotes (see [1] for discussion). The modification of Cs is achieved by the addition of a methyl group, a process referred to as cytosine methylation, or DNA methylation. In vertebrates, the methylation of Cs occurs in the context of a C followed by a guanine (G), also referred to as a CpG context [2], and has been shown to be important in gene regulation [3]. The study of methylation in modern genomes has thus become a powerful tool to understand processes such as aging [4], exposure to chemicals like lead [5], and cancer research [6].

One way to measure methylation is to use methods that distinguish between methylated and non-methylated Cs (non-mCs). The most common protocol used today to differentiate between these two C-states is bisulfite treatment, where non-mCs are deaminated to uracils (Us) (read as thymines (Ts) after PCR amplification) and mCs are left as Cs. The resulting C to T misincorporation when aligned to a reference genome is interpreted as a non-mC, while an unchanged C is inferred as a mC. Bisulfite conversion is an effective tool in modern DNA; however, it is a destructive process to DNA [7] and has therefore not been seen as optimal for ancient samples. Recently, a new protocol has been developed, the NEBNext® Enzymatic Methyl-seq Kit (EMseq), which has been shown to be effective for only picograms of DNA [8]. The EMseq protocol uses a two-step process of oxidation of mCs to protect them and an enzymatic deamination of unprotected non-mCs, which leads to the same C to T vs C to C differentiation between non-mCs and mCs as produced by the bisulfite conversion gold standard.

Ancient DNA (aDNA) is the study of post mortem DNA and poses a specific set of challenges. After death, various processes degrade the DNA of the organism, making it harder to extract and sequence. First, DNA is fragmented into ever shorter fragments [9]. This makes aDNA at best very short and often lacking sufficient DNA fragments that are long enough for meaningful analyses. The minute amounts of DNA also make aDNA susceptible to modern DNA contamination as well as leaving endogenous DNA at the risk of being overwhelmed by DNA from microbial sources that colonize the bones after death [10].

Despite these challenges, bisulfite treatment has previously been applied to aDNA samples. One of the first of these studies applied bisulfite treatment to ~ 26,000-year-old steppe bisons. The treated samples were then used as templates in a PCR to amplify four retrotransposon elements in an attempt to determine methylation measurements; however, only one of them had an amplifiable product [11]. A second study also tried to amplify specific regions of interest, in this case LINE-1 elements, in Native American samples after bisulfite treatment [12]. A more recent study bisulfite-treated tissue samples from two nineteenth century mummies and subsequently determined methylation levels using the Illumina EPIC BeadChip. They had low DNA concentrations which led to low signal intensities, but it was nevertheless possible to assign the known tissue of origin using methylation signals [13]. None of these studies used next generation sequencing technology and thus relied either on targeting long enough ancient fragments to be able to amplify regions of interest or bead chips designed for modern DNA. A more successful method of studying methylation in aDNA bioinformatically infers methylation from the natural deamination of Cs that occurs over time in aDNA. The rate of deamination increases toward the end of the fragments [14] and with the age of the sample [15]. The deamination of non-mCs turns the Cs into Us; however, the methyl group added during the methylation of Cs means that the removal of the amine group transforms Cs directly into Ts. aDNA can be treated with a combination of uracil deglycosylase and endonuclease VIII (USER treatment) to excise uracils and eliminate the C to T signal left by non-mCs leaving a C to T misincorporation signal caused only by the deamination of mCs [16]. Two main bioinformatic methods have been developed that take advantage of USER treatment to infer methylation levels in aDNA, RoAM [17] and DamMet [18]. RoAM has allowed for the study of methylation in Neanderthals and Denisovans, a sister group to Neanderthals. It has led to insights into morphology, brain disorders, and possibly even diet [17, 19–21]. DamMet was recently used to infer a methylation clock and castration status in ancient horses [22]. As the main source for C to T misincorporation is at the first and last few bases of a fragment, the overall rate of C to T misincorporation due to methylation is small, and accurate reconstruction of the premortem methylation requires a high coverage of the genome of at least 15 × on average [23]. This restricts this type of analysis to well preserved aDNA samples subjected to high-coverage shotgun sequencing or targeted capture of specific regions. Producing high-coverage ancient genomes is expensive in terms of capture and sequencing costs, although it has been recently shown that population DNA methylation patterns can be reconstructed from cohorts of samples sequenced to low depth [24]. Furthermore, methylation is measured in windows of consecutive CpG positions, lacking the ability to provide base-pair resolution as well as only detecting the more common 5-methylcytosines but not 5-hydroxymethylcytosines.

A direct examination of methylation through a methylation conversion treatment in aDNA would be invaluable to enable the study of more degraded samples and to measure methylation directly at base-pair resolution. Here, we examine two different methylation treatments in ancient samples: bisulfite treatment and the EMseq method. We find that the EMseq method, which we hypothesized would be a good candidate for aDNA due to its success with low input amounts, needs further optimizations due to biases that arise in one or both of its two-step conversion process. Meanwhile, bisulfite treatment, in combination with a single-stranded library preparation method used for aDNA, provides good performance and opens the door for direct measurement of aDNA methylation.

## Results

In order to test various methylation protocols, we chose two samples for which high-coverage data exists as part of the Allen Ancient Genome Diversity Project [25]. This high coverage data comes from the two samples, Zvej16 (I4438 from [26]) and SP75 (I3957, see Additional file 1 and [27]). The data come from double stranded libraries produced from cochlea powder DNA extracts with a USER pre-treatment to remove Us. The libraries were then shotgun sequenced to a depth of 28.98X and 28.53X, respectively. We produced additional extracts from the same cochlea bone powder for each of the two samples and applied a range of methylation methods (Tables S1 and S2).

Furthermore, we added data from 20 additional samples with varying ranges of DNA preservation for a subset of methods to increase our sample size (Additional file 1: Tables S1 and S2). Twelve of these additional samples come from six mummified human remains dating to the eighteenth to nineteenth century and curated at the Department of Anthropology, Hungarian Natural History Museum (HNHM) (see Additional file 1 and [28–39]). The samples include both a cochlea and a molar sample per individual. A lung tissue sample of a different mummified individual from the same crypt was previously bisulfite treated and described in [13]. The other eight samples come from cochleas and have produced low-coverage (less than twofold) shotgun sequencing data from USER treated libraries as described in [40].

### Bisulfite and EMseq methylation treatments

**Fig. 1:**
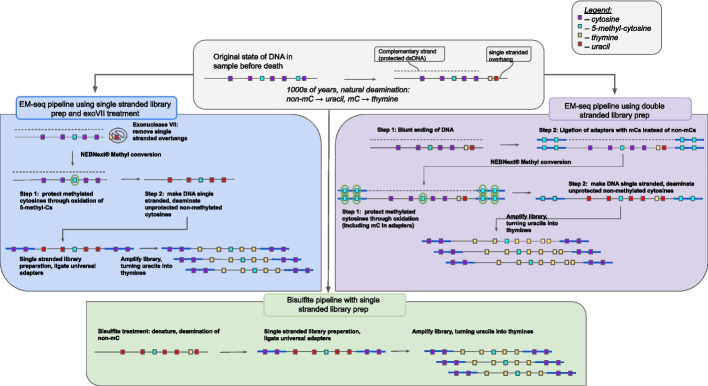
Schematic of the laboratory pipelines tested in this study. DNA extracts were methyl treated either with the EMseq methylation conversion (blue and purple boxes) or with the bisulfite methylation conversion (green box). EMseq was combined either with double-stranded library preparation methods (either the NEBNext Ultra II or a double-stranded method commonly used in aDNA analyses, purple box) or a single-stranded library method developed for aDNA (blue box). The three library prep methods were in addition repeated with a combination of an exonuclease VII pre-treatment, here only shown in the blue box. The bisulfite treatment was only combined with the single-stranded library preparation method (green box)

We applied a total of seven variations of pretreatments, methylation treatments and library preparation methods to extracts of both Zvej16 and SP75 (Fig. 1). For methylation treatment, we used either bisulfite treatment or EMseq. The EMseq kit from NEB (NEBNext® Enzymatic Methyl-seq Kit) comes with a double-stranded library preparation method and a methylation conversion module. We applied both their library preparation method and the EMseq conversion module with small changes detailed in the methods below. These libraries are subsequently referred to as NEB-EMseq. As the library preparation method included in the EMseq kit is not optimized for aDNA, we also replaced the NEB library preparation method with a double-stranded library method used in aDNA [41]. These double-stranded libraries, referred to as dslib-EMseq here, were then enzymatically treated with the conversion module part of the NEBNext EM-seq kit to convert non-mCs to Us as described above (see purple box of Fig. 1 for a schematic overview of the double-stranded libraries combined with the EMseq methylation conversion).

As the product of the methyl conversion is single-stranded, the double-stranded library preparation methods must precede the methyl conversion, necessitating the ordering of adapters with mCs instead of non-mCs. These adapters are expensive due to the number of mCs needed and drive up the cost of the method. Single-stranded library preparation methods are more sensitive to aDNA as they convert more fragments into library, and a new single-stranded method, the Santa Cruz Reaction (SCR) [42], has been published that is more cost effective and less time consuming than previous aDNA specific single-stranded methods [43, 44]. We therefore combined the SCR with the EMseq conversion module. However, instead of performing the library preparation first, we first converted the non-mCs into Us enzymatically using the EMseq conversion module, and then followed this conversion with the SCR. This means we can use the SCR protocol and adapters without any changes. We refer to this method as the sslib-EMseq method (see blue box in Fig. 1 for an overview).

aDNA shows an increase in C to T misincorporation the closer the base is to the end of the fragment [14]. This signal is driven by non-mCs and mCs deaminating at the ends of aDNA fragments [14]. As mCs deaminate into Ts directly, they will be indiscernible from the deaminated non-mCs after methylation conversion and could cause a bias in downstream analyses. While deamination has also been shown to happen within fragments due to gaps [45, 46], and can happen at blunt ends too, much of the deamination is driven by short single-stranded overhangs [46]. We wanted to remove as much of the possible bias of miscalling mCs due to deamination as possible by removing single-stranded overhangs. To do this, each of the three methods, NEB-EMseq, dslib-EMseq, and sslib-EMseq, were repeated again with a pre-treatment of the extract with exonucleaseVII (exoVII). The subsequent libraries are referred to as exoVII-NEB-EMseq, exoVII-dslib-EMseq, and exoVII-sslib-EMseq respectively.

Lastly, we replaced the EMseq method with bisulfite treatment. We used the EZ DNA Methylation-GoldTM Kit by Zymo Research on each of the extracts, without any changes in the protocol. The single-stranded product was then used as a substrate for the SCR [42] to prepare libraries. These libraries are subsequently referred to as bisulfite libraries.

The additional 20 samples from Additional file 1: Table S1 were treated with only a subset of the above variations: sslib-EMseq, exoVII-sslib-EMseq, and bisulfite (Additional file 1: Table S2).

Each of these libraries were indexed using a double indexing method [47] and a U-tolerant polymerase (Q5U from NEB). After amplification, sslib-EMseq and bisulfite libraries of each Zvej16 and SP75 were enriched for the methylome using the methylome capture from Twist Biosciences. We sequenced both the non-captured and captured libraries on the Novaseq 6000 system, producing 4–15 million reads per library.

### Positive controls

We used two types of positive controls to examine biases in various treatments. Our first set of positive controls were to test the effectiveness of exonuclease VII (exoVII) treatment. ExoVII has a 5′ to 3′ as well as a 3′ to 5′ exonuclease activity for single-stranded DNA, so treatment of aDNA fragments should fully remove the single-stranded overhangs and leave only the double-stranded fragments. We tested this with three hybridized oligonucleotide positive controls (see Additional file 1: Table S3). One positive control (regular exoVII) had a 60-bp strand hybridized to a 40-bp strand, allowing for one 10-bp overhang on each end. The second control (U-exoVII) had the 60-bp strand replaced by a sequence containing Us at the overhangs, while the third (mC-exoVII) had mCs in the overhangs. Each control was then sequenced with and without exoVII treatment. All of the controls showed that the exoVII treatment was incomplete, with 3- to 5-bp overhangs remaining (Additional file 1: Fig. S1). As it has been shown that single-stranded overhangs in aDNA are short (1–2 bp) [46], further optimization of this treatment is still needed.

Our second set of positive controls were to capture methylation treatment biases. The NEBNext® Enzymatic Methyl-seq Kit comes with two positive controls to be spiked into each sample before fragmentation: Control DNA CpG methylated pUC19 and Control DNA Unmethylated Lambda. After sequencing, these controls can be aligned to their respective reference genomes and the percent of mC in non-CpG context can be determined (see [8]). As the kit is designed for modern DNA and calls for fragmentation before treatment, the positive controls do not come fragmented and are thus not suitable for aDNA work. Instead, we designed two positive controls that mimic the controls from the kit but can be examined in more detail after sequencing. Each control is a double-stranded 60-bp oligonucleotide, one with all 15 Cs methylated (full-methyl-positive) and one with all 15 Cs unmethylated (no-methyl-positive) (Additional file 1: Table S3). Both controls were spiked into the extract before any treatment or library preparation.

For the full-methyl-positive, we expect none of the Cs to be deaminated after treatment. We see that the dslib (with and without exoVII treatment) as well as the non-exoVII treated NEB and the bisulfite treatment have mostly one C deaminated (Additional file 1: Fig. S2A-B), with only strand 2 showing a slight bias as to position (Additional file 1: Fig. S2C-D). Meanwhile, the other treatments show up to 7 Cs deaminated (Additional file 1: Fig. S2A). We expect the opposite result for the no-methyl-positive control, with all 15 Cs being deaminated. In strand 1, all treatments cause as few as 7 Cs to be deaminated, with the bisulfite treatment having the lowest amount of deaminated Cs at 15.7% (Additional file 1: Fig. S3A-B). Again, strand 2 shows a more distinct trend of a bias toward which position is less likely to be deaminated, although it is not consistent across treatments (Additional file 1: Fig. S3C-D).

While these controls cannot be directly compared to the results of the controls from the NEB kit, they do give insights into the effectiveness of methyl conversion in a control situation. Further investigation is needed to understand how CpG context, length, or the previous presence of Us could affect these controls.

### Effect of methyl treatment on DNA yields

**Fig. 2:**
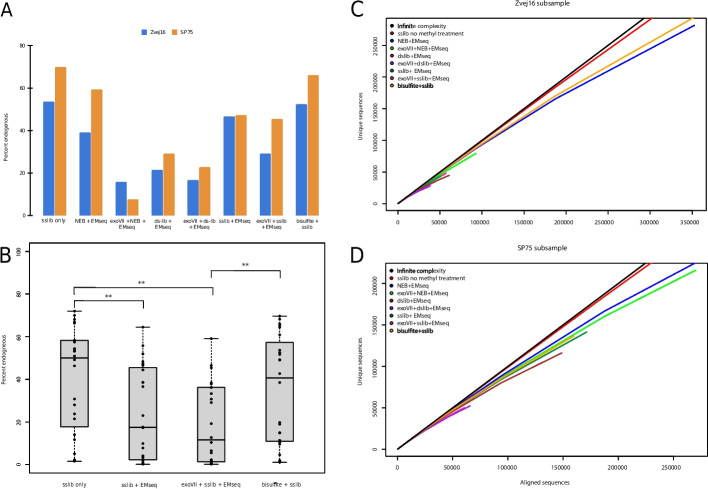
Effect of methyl treatment on DNA. **A** Comparison of the percent endogenous of Zvej16 and SP75 using the single-stranded library method with no methyl conversion, to the various methylation conversion and library preparation method combinations. **B** Comparison of the percent endogenous of all individuals included in this study using either the EMseq methyl conversion or the bisulfite conversion or neither conversion. Percent endogenous was calculated for each library by dividing the number of mapped reads with a mapping quality of 30 or greater by the raw reads. **C** Subsampling of duplicate removed (unique) sequences versus aligned sequences of Zvej16. The black line represents infinite complexity, followed by the sslib with no methylation treatment, bisulfite + sslib, NEB + EMseq, exoVII + NEB + EMseq, exoVII + sslib + EMseq, sslib + EMseq, dslib + EMseq, and finally exoVII + dslib + EMseq. Subsampling was done from 100 pg input into various combinations of methyl treatment and library preparation methods. **D** Subsampling of SP75. The black line again represents infinite complexity, followed by the sslib with no methylation treatment, NEB + EMseq, exoVII + NEB + EMseq, bisulfite + sslib, sslib + EMseq, dslib + EMseq, exoVII + sslib + EMseq, and finally exoVII + dslib + EMseq

In order to understand how destructive methylation treatment is to aDNA, we first examined the reduction in percent endogenous after methylation conversion in Zvej16 and SP75. When we compare the percent endogenous of each method to DNA extracts made into libraries using the SCR method with no methylation conversion, there is always a reduction of percent endogenous (2.9–61.4%), with the least reduction seen for the bisulfite treatment (2.9–3.0% bisulfite treatment vs. 8.8–61.4% other treatments) (Fig. 2A, Additional file 1: Table S4). As this only represents two samples, we treated additional aDNA samples, from fourteen cochleas and six molars, from various time periods with both the sslib-EMseq and bisulfite methods to increase our sample size to compare percent endogenous. These additional samples are younger than the previous two samples; however, they have a range of endogenous DNA levels indicating varying DNA preservation. Ten of these younger samples have lower percent endogenous than the two previous older samples (Additional file 1: Table S4), making them a good test set despite the younger ages. The difference between no methyl treatment and methyl treatment is only significant for the sslib-EMseq method and the exoVII-sslib-EMseq method (Wilcoxson rank sum test, *p* = 0.008 and *p* = 0.002, respectively) but is non-significant comparing no methyl treatment and bisulfite treatment (Wilcoxson rank sum test, *p* = 0.40). For each sample, bisulfite treatment has a higher percent endogenous (0.48 to 26.25%, average increase of 11.5%) than the sslib-EMseq method (Additional file 1: Table S4) although there is no significant difference between the two treatments (Wilcoxson rank sum test, *p* = 0.056) (Fig. 2B). The difference does become significant when comparing the bisulfite treatment to the sslib-EMseq method with an exoVII pretreatment (Wilcoxson rank sum test, *p* = 0.008). A comparison of percent endogenous and ng of DNA used as template for bisulfite treatment shows a slight relationship (*R*^2^ = 0.148, *p* = 0.064 (Additional file 1: Fig. S4)).

While percent endogenous is an indication of DNA preservation in aDNA, we wanted to understand the effect of the methylation methods on the complexity of the libraries, meaning how many unique library molecules are left after treatment and how much sequencing it takes to start seeing PCR duplicates. Both Zvej16 and SP75 are complex enough samples to produce above 28-fold coverage genomes, so sequencing the libraries deeply enough to see enough PCR duplicates to make inferences on complexity would be prohibitively expensive. Instead, we diluted both extracts to a concentration of 100 pg/µL and then used 1 µL (corresponding to 100 pg) as input for each method and subsequently sequenced each library to produce 10 million reads each. We then subsampled the aligned sequences and checked how many duplicates we see after each subsampling as was done in [48]. For both samples, the non-methyl converted sslib libraries had the highest complexity with the closest fit to infinite complexity (Fig. 2C, D). For Zvej16, the bisulfite method has the second highest complexity (Fig. 2C), while for SP75, the NEB EMseq treatments, both with and without exoVII treatment, have a higher complexity than bisulfite treatment; however, even in this sample, bisulfite has the fourth highest complexity (Fig. 2D). The percent endogenous of the 100 pg input is lower than the undiluted extracts (by 2.7% to 51.2%, see Additional file 1: Table S4) with again the bisulfite treatment having the highest percent endogenous, even at such low input amounts (Additional file 1: Fig. S5).

### Potential biases introduced during methylation treatment

**Fig. 3:**
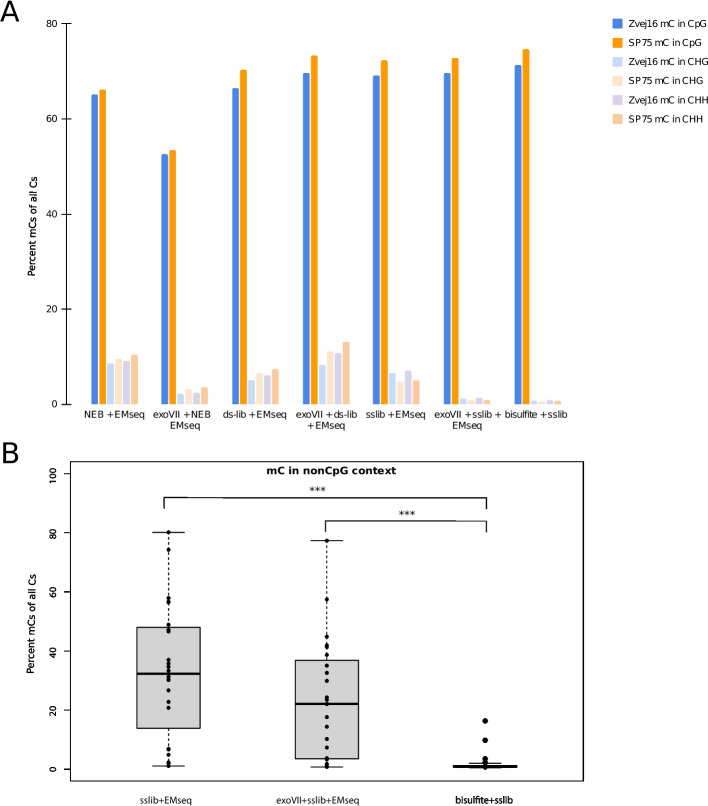
The percentage of methylated Cs of all Cs in various C-contexts. **A** Of Zvej16 and SP75 using various methylation treatments in both a CpG context (darker colors) and non-CpG contexts. H refers to a base other than G. **B** Of all samples used in this study comparing the EMseq method with and without exonuclease VII treatment in combination with the single-stranded library method as well as bisulfite treatment. This comparison is restricted to non-CpG contexts. The two outliers in the bisulfite treatment data are Vác 193 Molar and Vác 164 Petrous

To assess the efficiency of each method in converting non-mCs to Us while not converting mCs to Us, we first examined the percentage of mCs detected bioinformatically in CpG contexts and non-CpG contexts. In mammals, we expect the number of mCs in non-CpG contexts to be low as methylation outside CpG is rare in mammals [49]. Previous studies have found the percentage to be below 0.6% [8]. Meanwhile, the average percent of mCs in CpG context is around 70–80% [50]. When examining the CpG contexts of Zvej16 and SP75, all methods involving the EMseq method, except the exoVII-sslib-EMseq method, have above 1% of the Cs in non-CpG contexts defined as mCs (Fig. 3A, Additional file 1: Table S5). This is most likely caused by an issue in the second step of the methylation conversion of the EMseq kit, where non-mCs are not being deaminated and are thus remaining Cs and are subsequently defined as mCs bioinformatically. The bisulfite conversion has the lowest mC percentage in non-CpG context (0.7–0.8%) as well as having a 71.4–74.7% mC in CpG context (Fig. 3A, Additional file 1: Table S5 and S6).

As both the exoVII-sslib-EMseq and bisulfite method had the lowest mC in non-CpG context, we examined if this result is also observed in additional samples. Thus, we tested the exoVII-sslib-EMseq, sslib-EMseq and bisulfite libraries from the 20 additional aDNA samples (Additional file 1: Table S2) for mC in various contexts and found that the exoVII-sslib-EMseq is not effective at reducing mC in non-CpG contexts for every sample. In fact, two samples, Vác 179 and 11,118, exhibit a percentage of mC in non-CpG contexts over 30%. Meanwhile, bisulfite treatment shows a consistent percent of mC in non-CpG context at or below 2.5% for all but three samples tested (Fig. 3B). The samples with greater than 2.5% mC in non-CpG context also have some of the lowest percent endogenous, which makes sense as percent endogenous correlates significantly with percentage of mCs in non-CpG context (*R*^2^ = 0.229, *p* = 0.018, Additional file 1: Fig. S6). In addition, the difference between the EMseq and bisulfite, as well as EMseq combined with exonuclease VII treatment and bisulfite treatment, is significant (Wilcoxson rank sum test, *p* = 4.3e − 08 and *p* = 5.9e − 07, respectively) (Fig. 3B). Interestingly, when examining this result for the 100 pg input for Zvej16 and SP75, we see a percentage of below 4% for mCs in non-CpG contexts for all treatments except using the dslib-method (Additional file 1: Fig. S7). This suggests that low input amounts of DNA are not the only cause for the high mC percent in non-CpG contexts in the additional samples.

Conversely, when examining the percentage of mC in CpG context in SP75 and Zvej16 across all treatments, the NEB method with the exoVII pre-treatment has 52.6–53.4% of mC in CpG context (Fig. 3A, Additional file 1: Table S6), almost 20% less than expected, indicating that for this method, there is potentially an additional issue with not oxidizing mCs, so mCs are deaminated in the following step of the conversion module and are thus defined as non-mCs bioinformatically. The same low percentages can be seen when examining the additional 20 samples, where the sslib-EMseq of one sample, 11,112, has a mC percentage of 30.2% in CpG context (Additional file 1: Fig. S8, Additional file 1: Table S6). The three samples with the highest percentage of mC in non-CpG context were also the three with the lowest percentage of mC in CpG context after bisulfite treatment, indicating a possible relationship between these two biases (Fig. 3B, Fig. S8 and Tables S5, S6).

To examine additional bias sources, we calculated the percentage of mC within and outside of CpG positions of CpG islands in the Zvej16 and SP75 samples across all treatment types. CpG islands are regions of DNA characterized by a high frequency of CpG sites and are often associated with gene regulatory elements. The methylation of Cs in CpG islands is expected to be low, even in CpG context [2]. We see 42.4–60% higher mCs outside CpG islands than within, consistent with a reduction in mCs in CpG context in CpG islands. However, the values are variable, with the exoVII-NEB-EMseq having the lowest (10.7% and 8.8% for Zvej16 and SP75 respectively) and bisulfite libraries having the highest (24.5% and 28.1% for Zvej16 and SP75 respectively) percent of mCs in CpG islands, 10.6–14.6% higher than two modern bones that were also bisulfite treated (Additional file 1: Fig. S9A and Additional file 1: Table S7). When examining a subset of treatments in the additional 20 samples from Additional file 1: Table S1, the bisulfite treatment had the most consistent results, concordant with the bisulfite values for Zvej16 and SP75. The EMseq in combination with the sslib method however had a large variation in values (Additional file 1: Fig. S9B), which is possibly due to the biases in methylation calling due to CpG context.

A third bias we examined is the effect of each treatment on read lengths. When comparing the length distributions of various treatments and the single-stranded library method without methylation conversion, we find that all methylation conversion methods bias toward longer fragments. The least biases occur in the treatments involving the NEB library preparation method and the EMseq method combined with the single-stranded library method and exoVII pre-treatment (Additional file 1: Fig. S10A). This indicates that methylation treatment, be it EMseq or bisulfite, is the most destructive to short aDNA fragments and would be least effective for samples with extremely short fragments. We further examined the length bias from bisulfite treatment using additional aDNA samples (Additional file 1: Table S2). Again, the bisulfite treatment causes a bias for longer sequences compared to libraries from the same samples that were not bisulfite treated (Additional file 1: Fig. S10B).

### Comparison to modern bone and high coverage data

Both the sslib-EMseq and the bisulfite libraries for Zvej16 and SP75 were sequenced further to deeper coverages (0.46X and 0.61X for the EMseq and 0.27X and 0.29X for bisulfite respectively). In addition, we captured each of these four libraries using the methylome capture kit from Twist Biosciences, sequencing for another 2.8–14.6 million reads. As DNA methylation patterns are tissue specific [51], we compared the methylation rates, hereafter referred to as beta values, of ancient bone samples with modern osteoblast methylation data produced from a 30-fold coverage bisulfite-treated sample [51] as well as methylation data from two modern bones [20]. In addition, we compared our Zvej16 and SP75 data to inferred methylation rates using RoAM and DamMet from high coverage non-bisulfite-treated data [17, 18].

**Fig. 4:**
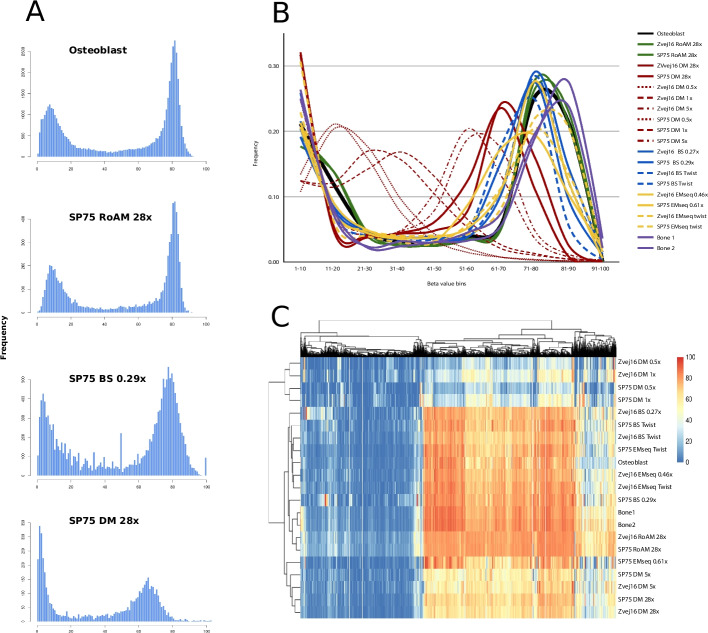
Beta value comparisons of chromosome 1. DM = DamMet, BS = bisulfite. Coverage for shotgun data is shown in the legend. Data produced by DamMet and RoAM are using high coverage USER treated data produced of both Zvej16 and SP75 as part of the Allen Ancient Genome Diversity Project. BS and EMseq data of Zvej16 and SP75 were produced from extractions made for this study of the same cochlea as the Allen Ancient Genome Diversity Project and methyl treated either using bisulfite treatment or EMseq treatment in combination with single-stranded library preparation. Twist captured data of the methylation treated libraries is also included. Bone1 and Bone2 are two modern whole bone samples bisulfite-treated and sequenced in [20]. **A** Histograms of beta values of Osteoblast data and data from SP75 using RoAM and DamMet to infer beta values from 28-fold data of USER treated data as well as the low-coverage sequencing of bisulfite (BS) treatment. **B** The frequency of beta values that fall into bins of 1–100 in increments of 10. **C** Clustered heatmap of beta values of chromosome 1. The legend shows the beta values with red being a beta value of 100 and blue being a value of 0. https://www.ebi.ac.uk/ena/browser/view/PRJEB71420. 2024

After segmenting the data based on the osteoblast beta values, we compared the variation in the histograms of the frequency of beta values for each of our comparisons (Fig. 4A, see Figs. S11-S27 for additional individual histograms). The osteoblast data has 80% of its beta values falling either below 20 or between 71 and 90, in a bimodal distribution of values (Fig. 4A, B, Additional file 1: Table S8). Both the RoAM beta inference, the low-coverage shotgun data and methylome capture of Zvej16 and SP75 show a similar distribution. An exception is the low-coverage data from SP75 using the EMseq method, where a higher frequency of beta values falls into the 61–70 bin than the 81–90 bin, broadening the peak (Fig. 4B, Additional file 1: Table S8). The high-coverage DamMet beta inference has a shifted peak with 21–23% of the beta values falling into the 61–70 bin. We examined the inferred beta values of both samples with DamMet using the high coverage data but downsampled to 0.5-fold, onefold, and fivefold coverage to better compare to our < onefold coverage data using the bisulfite and EMseq methods. As the coverage is reduced, the peak also migrates to the left, with the beta inference using DamMet on the 0.5-fold subsampling of the high-coverage data having 20–21% of its beta values fall within the 11–20 bin. Interestingly, the modern bone data (Bone1 and Bone2), which comes from whole bones and not a single cell type, has a slightly shifted peak for higher methylation rates (Fig. 4B, Additional file 1: Table S8).

In order to understand the relationship between the beta values of our various comparisons, we clustered the beta values using a clustered heatmap (see the supplementary materials) (Fig. 4C). The heatmap clusters the DamMet inferred beta values of the onefold or lower subsampled high-coverage data into a separate group. The remaining DamMet inferred beta values cluster with the shotgun EM-seq data from SP75, which corresponds to the shifted beta value frequency shift described above. The third main cluster contains the rest of the comparisons, with a subgroup forming containing the RoAM beta value inferences, both modern whole bones and the SP75 bisulfite treatment shotgun data (Fig. 4C).

Lastly, we examined the potential effect of naturally deaminated Cs in ancient DNA. Naturally deaminated non-mCs deaminate to Us, which should not cause any biases; however, mCs deaminate directly to Ts and will then be interpreted as non-mCs bioinformatically after bisulfite treatment. We determined the rate of C (in the reference genome) to T (in the sequenced reads) substitution inferred to have been caused by natural deamination of both mCs or non-mCs in the non-bisulfite high coverage data from both SP75 and Zvej16 (Additional file 1: Table S9 and S10, last column). The high coverage data was produced from libraries that were partially uracil-DNA-glycosylase (UDG) treated and thus have a reduced C to T signal at the very first and last base [52]. When we filter the high coverage reads for positions that overlap with the bins of beta values determined from the bisulfite-treated and Twist captured data, and only examine CpG positions, we see an increase in the C to T rate as the methylation rate increases (Additional file 1: Fig. S28A). This effect can be seen even in the substitution rate by methylation rate at the first position of the read when comparing the rate of at all Cs versus Cs at non-CpG positions (Additional file 1: Fig. S28B).

## Discussion

Our results show that aDNA can be effectively treated to convert non-mCs to Us to directly measure methylation rates, using two conversion methods developed and perfected for modern DNA. We tried a variation of library preparation methods and conversion methods to understand how effective they are for aDNA. Based on the reduction in percent endogenous, the DNA sequence complexity, and the lack of detectable biases in uracil conversion, we find that bisulfite treatment, in combination with an aDNA specific single-stranded library preparation method [42], is the most promising pipeline to assess direct methylation rates in aDNA. Even at low-coverage (below onefold), this combination of methods performs as well as the previous bioinformatic methods applied to high coverage (over 28-fold) data. However, such low coverage should be used with caution for future aDNA methylation studies. Even in modern DNA, a minimum of 5X to 15X has been recommended for whole genome sequencing [53]. We recommend careful deliberation of what coverage would be needed as it is dependent on the question asked, whether capture or whole genome sequencing is used, the number of samples required for the study, and the sample preservation.

Bisulfite treatment has been used to examine methylation in aDNA in previous studies, usually relying on the use of primers to amplify regions of interest after bisulfite treatment [11, 12]. A more recent study [13] examined the methylation signal of a lung tissue sample taken from another mummified individual from the same crypt as the six mummified Vác individuals used in this study (Additional file 1: Table S1). This study included an additional mummified sample from Switzerland and used an approach where they determined methylation based on the Illumina EPIC methylation microarray after bisulfite treatment. They prepared double-stranded libraries from non-bisulfite-treated extract and had a percent endogenous of 0.65% for the Vác sample after sequencing, indicating a poorly preserved sample. As their data has been produced with a different method and from a different tissue, and methylation has been shown to be heavily tissue dependent [51], and both their data set and ours is small, it is difficult to compare the two datasets.

While bisulfite treatment is the most promising pipeline shown in our study, and has had success in previous aDNA studies, albeit with different methods, additional work should be conducted to optimize the EMseq method for aDNA. The EMseq method is a recent method and has quickly become a popular method to use for methylation treatment in modern DNA. It strives to reduce biases in GC content and mapping rates seen due to bisulfite treatment [54]. Studies have shown that the EMseq method has fewer biases in GC content and fragments DNA less than bisulfite treatment [8, 55]. However, this is dependent on which bisulfite kit and which library preparation method are used, as bisulfite treatment in combination with a different single-stranded library preparation method has been shown to be almost as effective as EMseq, especially for potentially degraded DNA [56].

In our aDNA samples, we show that bisulfite treatment introduces a size bias toward longer fragments, while EMseq combined with the NEB library preparation method reduces the bias toward longer fragments. The biggest hurdle for the NEB-EMseq method is that it introduces biases in non-CpG contexts, most likely occurring due to a reduced efficiency in the second step of the conversion module, where the APOBEC3A enzyme mixture is not deaminating non-mCs effectively. This, in turn, leaves the non-mCs as Cs, which are then assigned as mCs during bioinformatic analyses. This bias is especially surprising, since work on modern plants has shown that the EMseq method reduced this signal compared to bisulfite treatment [55]. One explanation may be that the EMseq method is geared toward a fragment size of 240–290 bp and at least 10 ng; thus, additional optimization is needed to more effectively deaminate non-mCs in this step. While optimizing the EMseq method for aDNA would be of use to increase the options for methylation conversion in paleoepigenetics, the EMseq method combined with the NEB library preparation method included in the full kit is 3.5 times more expensive than the EZ DNA Methylation-GoldTM Kit we used for bisulfite treatment in combination with the SCR single-stranded library preparation method, a cost consideration that is not negligible.

Cs in aDNA deaminate naturally, causing different reactions for mC and non-mCs. Natural deamination of mCs turns them directly into Ts, which in turn causes these mCs to be bioinformatically read as non-mCs. Deamination is most prevalent at fragment ends, and methylated Cs have been shown to have a deamination rate similar to non-mCs (see Additional file 1: Fig. S5 in [48]), which can cause a bias when calling mCs from bisulfite-treated data from aDNA. Areas with higher methylation rates are then more affected by this bias, as we see using partially UDG-treated libraries. Optimization is thus needed to better understand and address the potential biases that could arise from this signal. In the laboratory, this would, for instance, include additional work on the use of exoVII to completely remove single-stranded overhangs. However, deamination has been shown to also occur internally in aDNA fragments in addition to the first few bases of blunt ends [46]. Furthermore, the removal of single-stranded overhangs shorten the already short aDNA fragments and, like USER treatment, should be considered carefully on a sample-by-sample basis as such fragment shortening could significantly affect DNA yields. An alternative approach would be to explore bioinformatic strategies to estimate and account for this bias, using multiple samples with non-bisulfite-treated and fully UDG-treated libraries as well as bisulfite-treated data to infer methylation biases and rates. Furthermore, refining current bioinformatic programs designed for modern methylation sequences to be more sensitive to aDNA sequences would also be of value.

## Conclusions

We describe here the most comprehensive study of direct methylation detection in aDNA. Of the methods tested, we find that bisulfite treatment combined with an aDNA specific single-stranded library preparation method is the least destructive to aDNA and can be applied to a multitude of aDNA samples. This method opens the door to a comprehensive study of methylation and subsequent examination of differentially methylated regions in ancient populations, which will provide an additional layer of understanding of various environmental effects such as diet, exposure to diseases and toxins, gestational effects, and bone diseases such as osteoporosis.

## Methods

### Laboratory pipelines

#### Bone grinding and DNA extraction

Cochleas and roots from molars from samples listed in Additional file 1: Table S1 were ground in a dedicated clean room as described in [57]. Fifty to 100 mg of bone powder was then used to extract DNA using the method from [58] to produce 50 µL of DNA extract. Extracts were measured on the Qubit3 with high sensitivity reagents for double-stranded DNA to assess the ng/µL concentration. Concentrations of extracts can be found in Additional file 1: Table S1. For samples Zvej16 and SP75, an aliquot of each extract was diluted to 100 pg/µL to allow for 100 pg input material for subsampling analyses.

#### EMseq

The NEBNext® Enzymatic Methyl-seq Kit (EMseq) is made up of two relevant parts: one is the library preparation (explained in more detail in the next section), and the other is the methylation conversion module (NEBNext® Enzymatic Methyl-seq Conversion Module – this module can also be ordered as a standalone kit). The EMseq conversion was performed as described in the “Protocol for use with Standard Insert Libraries (370–420 bp)” (NEB #E7120) with the following changes. (1) Neither of the supplied positive controls (Control DNA CpG methylated pUC19 and Control DNA Unmethylated Lambda) was used as they do not come sheared and could not be effectively sheared in our cleanroom. (2) After oxidation, DNA was cleaned up using Minelute columns (Qiagen) by binding to the column with 250 µL of buffer PB, washing once with 750 µL of wash buffer PE and eluting in 20 µL of buffer EB. (3) Subsequent denaturation was performed by heating the oxidized and cleaned DNA to 95 °C for 3 min followed by an ice water bath for 3 min. Samples were kept on ice as recommended in the manual. (4) The final deaminated DNA was cleaned using Minelute columns (Qiagen), with 500 µL of buffer PB to bind and eluting in either 40 µL of EBT (Qiagen buffer EB with 0.05% tween) if combined with either of the double-stranded protocols or in 20 µL of EBT if the DNA was then input for the single-stranded protocol.

#### Bisulfite treatment

Between 0.1 and 10.6 ng of DNA (corresponding to 1–10 µL) of DNA extract was used as input for bisulfite treatment. The sample was filled to 20 µL using EBT as recommended by the manual. The EZ DNA Methylation-GoldTM Kit by Zymo Research was used for bisulfite treatment. The protocol was followed exactly with no deviations. After elution, the 10 µL of eluate were filled to 20 µL of by adding 10 µL of EBT to prepare for the single-stranded library preparation.

#### NEBNext UltraII library preparation as part of the EMseq protocol

Between 0.1 and 9.2 ng of DNA (corresponding to 1–5 µL) of DNA extract was used as input for the NEBNext UltraII library protocol. The protocol and reagents used were part of the NEBNext® Enzymatic Methyl-seq Kit. We used the Protocol for use with Standard Insert Libraries (370–420 bp) (NEB #E7120) with the following changes. (1) We did not include the supplied positive controls (Control DNA CpG methylated pUC19 and Control DNA Unmethylated Lambda). (2) DNA was not fragmented as ancient DNA is already naturally fragmented. (3) The DNA extract was filled up to 50 µL using EBT for the End Prep. (4) Adapter-ligated DNA was cleaned using a Minelute column (Qiagen) by binding the DNA with 468 µL of buffer PB and eluting in 28 µL of EBT.

#### Double-stranded library preparation

Between 0.1 and 9.2 ng of DNA (corresponding to 1–5 µL) of DNA extract were used as a template for double-stranded library preparation. We followed the protocol outlined in [41], with the following changes: (1) adapters from [41] were ordered to contain mCs instead of Cs to avoid them being deaminated in the methyl conversion, and (2) in the adapter fill-in step, the regular dNTPs were replaced with dNTPs that contain a d-methyl-CTP instead of a dCTP.

#### Single-stranded library preparation

After either EMseq methyl conversion or bisulfite treatment, 20 µL of converted DNA was used as input for the Santa Cruz Reaction as outlined in [42]. No changes were made from the protocol outlined in the supplementary materials of the paper. As the DNA after methyl conversion is single-stranded, we could not measure the concentration on the Qubit. Instead, we calculated the appropriate SSB, P5, and P7 concentrations using the concentration of the DNA extract before methyl conversion minus 20%.

Between 0.1 and 9.2 ng of DNA (corresponding to 1–5 µL) of DNA extract were also used for each sample to produce single-stranded libraries without any methyl conversion.

A description of what sample was treated with which combination of methods can be found in Additional file 1: Table S2.

#### Exonuclease VII testing and treatment

In order to reduce potential biases from natural deamination on single-stranded overhangs of ancient DNA, we tested using exonuclease VII (NEB M0379S) to remove single-stranded overhangs as this enzyme has both 5′–3′ and 3′–5′ exonuclease activity. First, we ordered multiple complementary oligonucleotides (Additional file 1: Table S3). These were then hybridized in the following combinations: exovii-1 and exovii-2 as a standard test, exovii-3 and exovii-2 to test Us in the overhangs, and exovii-4 and exovii-2 to test mCs in the overhangs. Hybridization occurred by combining 10 µL each of 20 µM concentrated oligonucleotides, 2.5 µL of nucleotide-free water,and 2.5 µL of 10 oligo hybridization buffer (0.5 M NaCl, 0.01 M Tris–HCl, 0.001 M EDTA) and heating to 95 °C followed by a cooling to 12 °C at − 0.1 °C/s. Each hybridized set was then treated with exonuclease VII by adding 10 µL of 5X exonuclease VII reaction buffer, 0.2 µM of the control oligonucleotide, 0.5 µL of 10 U/µL exonuclease VII, and filling to 50 µL with nuclease-free water. Both exonuclease VII and non-exonuclease VII-treated controls were subsequently made into libraries using the single-stranded preparation described above and subsequently indexed and sequenced as described below.

To test exonuclease VII treatment on our samples, Zvej16 and SP75 extracts were treated with exonuclease VII as described above. After MinElute column cleanup, the eluate was then used as input for each of the three library preparation protocols and EMseq combinations.

#### Indexing PCR and quality control

After library preparation and methyl conversion were complete, 1 µL of a 1:40 dilution of each library was measured via qPCR using the IS7 and IS8 primers from [44] and 2X Biozym Blue S´Green (Biozym) master mix. After qPCR, the number of copies per sample was measured as well as the number of ideal cycles to amplify without amplifying into plateau (see [44]).

One fourth of each library was then amplified in an indexing PCR using Q5U (NEB) and unique indexes for both the P5 and P7 ends ([44] for list of possible indexes). PCR reactions were performed for various cycles depending on the cycle number calculated during the qPCR. After indexing, the libraries were cleaned using NucleoMag NGS Clean-up and Size Select beads (Machery-Nagel) at a 1.2-fold concentration. Samples were eluted in 20 µL of EBT, measured on the Qubit3 and the Tapestation.

#### Twist capture

After the indexing PCR, Zvej16 and SP75 libraries (see Additional file 1: Table S2) were captured using the Twist methylome capture (Twist Biosciences, Twist methylome V1 TE-96341190). First, 3 µL of each indexed library was re-amplified using KAPA HiFi 2 × MasterMix (Roche) and IS5 and IS6 primers ([41]) for 20 cycles. Reactions were cleaned using the NucleoMag NGS Clean-up and Size Select beads at 1.8-fold concentration. Cleaned amplifications were then dried using a Speedvac for 1 h at room temperature. After drying, the Twist Biosciences protocol was followed for capture using a modified version for aDNA [59]. After capture, samples were again amplified using KAPA HiFi 2 × MasterMix and IS5 and IS6 primers for 20 cycles, cleaned using a 1.8-fold concentration of NucleoMag NGS Clean-up and Size Select beads, and measured on the Qubit3 and Tapestation to assess quality.

#### Positive controls

As the EMseq protocol comes with positive controls (Control DNA CpG methylated pUC19 and Control DNA Unmethylated Lambda) that are unfragmented, they are not suitable for the cleanroom. Instead, we designed our own positive controls: a fully methylated and an unmethylated control (Additional file 1: Table S3). Both controls were hybridized as described above in the exoVII section, and then pooled together. Each extract had 0.4 µL of the 0.1 µM positive control pool added to the sample input before any treatment took place.

#### Sequencing and demultiplexing

Each library was added to sequencing pools to allow for ~ 5 million sequencing reads, unless deeper sequencing was desired; then, up to 15 million reads were sequenced. Pools were sequenced on the NovaSeq 6000 on an XP SP 100 SR cycle flowcell at the Vienna BioCenter Core Facility (VBCF). After sequencing, the VBCF performed basecalling and demultiplexing and sent us demultiplexed fastq files.

### Bioinformatic pipelines and analyses

#### Mapping and first quality controls and filters

Demultiplexed files had adapter sequences and reads shorter than 30 bp removed using cutadapt 4.2 [60]. For mapping, we modified the hg19 genome reference using the bismark_genome_preparation package from Bismark [61]. After adapter removal, sequences were mapped to the modified reference genome using Bismark [61] with the single end read parameter. After mapping, the percent endogenous of each library was calculated by dividing the number of mapped reads with a mapping quality of 30 or greater by the raw reads. Duplicates were removed using the samtools rmdup package [62]. Duplicate removed reads with a map quality of ≥ 30 were kept for further analyses. Bismark produces multiple output files, one of which calculates the mapping percent and the percentage of mCs in CpG contexts as well as various non-CpG contexts. Differences in CpG-contexts and percent endogenous were calculated using the Wilcoxon rank sum exact test using R 4.3.1 [63] in Rstudio 2023.06.1. Correlations to produce *R*^2^ values were calculated using the correlation function, and associated *p*-values were determined using a linear regression model using R 4.3.1 [63] in Rstudio 2023.06.1 to correlate percent endogenous to ng of input and percent endogenous to percent of mC in non-CpG contexts.

Non-methyl treated libraries were mapped to the hg19 reference genome, as is standard in aDNA work, using BWA [64] with the parameters -n 0.01 -o 2 -l 16,500. After mapping, the percent endogenous of each library was calculated by dividing the number of mapped reads with a mapping quality of 30 or greater by the raw reads. Duplicates were removed using the samtools rmdup package [62]. Duplicate removed reads with a map quality of ≥ 30 were kept for further analyses.

#### Complexity

For both Zvej16 and SP75, we input 100 pg of extract for each of the treatments. These samples were subsequently shotgun sequenced to produce ~ 10 million reads. In order to infer complexity, these reduced input samples were filtered as above except duplicates were not removed. Instead, the samtools -s option was used to subsample the original bam file reads to produce new bam files with 350 reads and sequentially doubling the read number to 1,500,000 reads. Each new bam file then had duplicates removed, and the number of unique (duplicate removed) and total reads were plotted using R 4.3.1 in Rstudio 2023.06.1 using the plot function.

#### Exonuclease VII controls

After sequencing the exonuclease controls, the sequences of both strands of each control (Additional file 1: Table S3) were extracted from the raw fastq files, and the length distributions of each control was calculated. The frequency of each length was then calculated by dividing the number of reads of each length by the total reads.

#### Positive controls

Positive control reads were extracted from each demultiplexed sample by searching for the correct sequence of both the top and bottom strands (Additional file 1: Table S3) in the raw fastq file. The number of reads that match both the top and bottom strands of each of the two positive control types were counted, and the percentage of positive controls of the total raw reads was calculated. The values ranged from 0.3 to 10%. For the full-methyl positive control, each strand has 15 positions of the 60 total that are mCs (and no non-mCs). The number of methylated Cs was counted as well as which position was methylated. For the non-methyl positive, each strand had 15 positions of the 60 total that are non-mCs (and no mCs). Again, the number of methylated Cs was counted as well as which position was methylated.

#### Length distributions

The length distributions of the filtered reads (see above) of each treatment possibility (Additional file 1: Table S2) of SP75 and Zvej16 were calculated from the final bam files. The frequency of length distributions was then calculated by dividing the number of reads of each length by the total reads. Length frequency distributions were calculated from all samples (Additional file 1: Table S2) in the same manner. The mean and standard deviation were then calculated using the dplyr and tidyr packages and plotted using a ribbon plot in ggplot in R 4.3.1 and Rstudio 2023.06.1.

#### Beta calling

Osteoblast data: We downloaded the bigwig file from [51] and extracted the beta values as well as chromosome and position data using R 4.3.1 and Rstudio 2023.06.1. These values were merged into a bed file.

RoAM data: We used the RoAM pipeline with default parameters [20] to produce genome-wide reconstruction of premortem DNA methylation of high coverage bam files provided by the Allen Ancient Genome Diversity Project (Zvej16 (I4438 from [26] at 28-fold coverage and SP75 (I3957) at 28-fold coverage).

DamMet data: To calculate *F* (inference of a beta value), we used DamMet [18] on the high coverage bam files provided by the Allen Ancient Genome Diversity Project (Zvej16 (I4438 from [26] at 28-fold coverage and SP75 (I3957) at 28-fold coverage) using a CpG window size of 20. We then downsampled both samples to 0.5, 1, and fivefold coverage using the samtools view -s option and re-ran DamMet with the same parameters.

EMseq and bisulfite data: We used the bismark_methylation_extractor package of Bismark [61] to extract the methylation information of each C-position. We use the methylation percentage as a beta value for each position.

As Bismark outputs a percentage, we convert all other beta measurements or approximations to percentages to allow for comparisons.

#### CGI controls

As we expect methylation rates to be lower in CpG islands (CGIs), we calculated the methylation rate within and outside of CGI regions for each of our treatments for SP75 and Zvej16 (Additional file 1: Table S7). To do this, we downloaded the cpgIslandExt track from the UCSC Genome Browser and intersected these positions where either a mC or non-mC (or both) had been called using Bismark using bedtools intersect [65]. We then calculated the percentage of mC within and outside of the CGI regions. For Bone1 and Bone2 from [20], we repeated the same analysis for each bone sample and included the data as a bisulfite-treated sample.

Subsequently, we included the additional 20 samples with bisulfite, exoVII-sslib-EMseq, and sslib-EMseq treatments (Additional file 1: Table S2) and compared methylation rates within and outside of CGIs. We calculated the mean and standard deviation using R 4.3.1 and Rstudio 2023.06.1 and then plotted the mean with standard deviation using ggplot.

#### Segmentation of data

We used the R 4.3.1 program methSeg from methylKit [66] to segment the osteoblast file into segments based on their methylation profile with the join.neighbor function turned on to allow neighboring segments that cluster into the same segmentation group to be joined into one segment.

In order to compare beta values for these segments, we used the positions of the segments to filter the other files (RoAM, DamMet, EMseq, and bisulfite beta value outputs). Each file was filtered for the positions that fall into the segments determined above, and the average beta value per segment was calculated.

#### Beta comparisons

To make comparisons computationally more manageable, we restricted the beta comparisons to chromosome 1. Segmented data from chromosome 1 from each type of treatment and analysis was combined with the segmented osteoblast data into one file. Data was visualized as a histogram using the hist function in R 4.3.1 in Rstudio 2023.06.1. We further calculated the fraction of beta values that fall into each size bin from 1 to 100 in bin-increments of 10. Clustered heatmaps of beta values for all samples were made using the pheatmap 1.0.12 function in R 4.3.1 and Rstudio (https://CRAN.R-project.org/package=pheatmap).

#### C to T misincorporation analysis

We filtered the high coverage bam files provided by the Allen Ancient Genome Diversity Project (Zvej16 (I4438) and SP75 (I3957)) for reads that map to chromosome 1. As these samples were partially UDG-treated and thus have reduced damage signals left at the very first and last base of each read [52], we calculated the C to T rate at the first 20 bp of all reads. We then used the beta value bins of the Twist capture of the bisulfite-treated data from the previous section and filtered the high coverage non-bisulfite data from chromosome 1 for each of the bins. Subsequently, we calculated the substitution rates at CpG positions and non-CpG positions for the first 20 bp of the 5′ ends of the reads that overlap each beta value bin (Additional file 1: Table S9 and S10).

## Supplementary Information

Additional file 1: Improved detection of methylation in ancient DNA Supplementary Materials. This file contains site descriptions of sites that have not been described in previous genomic studies as well as supplementary tables and figures.

Additional file 2: Review history.

## Data Availability

The datasets generated and analyzed during the current study are available as raw fastq files in the European Nucleotide Archive (ENA) under accession number: PRJEB71420 [67].
