# Supplementary material for: Improved detection of methylation in ancient DNA
Source: Genome Biol. 2024 Oct 10;25:261. doi: 10.1186/s13059-024-03405-5 (PMC11465500; doi:10.1186/s13059-024-03405-5)
Supplement: Supplementary file 1 — Additional file 1: Improved detection of methylation in ancient DNA Supplementary Materials. This file contains site descriptions of sites that have not been described in previous genomic studies as well as supplementary tables and figures. [file 13059_2024_3405_MOESM1_ESM.docx]

Improved detection of methylation in ancient DNA

Supplementary Materials

# Sample descriptions

We include a total of 22 samples from 16 individuals in this study (Table S1). Zvej16 (I4438) has been described in [[26 (reference numbers from main text)]](https://paperpile.com/c/jcKi2X/kotD). In addition, samples 10658, 3543-3547, 2050, 11112 and 11118 have been described in [[39]](https://paperpile.com/c/jcKi2X/3dvyq) under the names R10658, R3543-R3547, R2050, R11112 and R11118.

**The SP75 individual from Sabinka 2 (Coordinates 53,11 на 91,20):**

The Sabinka 2 burial ground is located near the villages Sabinka and Novokurskoe, Beisk district, Khakassian republic, Russia. The site was excavated by P.Pavlov in 1984-1985. The burial ground was identified as belonging to the Karasuk culture of the Late Bronze Age. Although characteristic Karasuk items were found in the vast area from Kazakhstan to Altai-Sayan region, the focal area of the culture was the Khakass-Minusinsk Basin.

The Karasuk burial grounds (kurgans) consist of square, less often round, stone fences. Single or double burials were made in stone boxes made of thin slabs, deepened to 1m. The deceased were placed stretched out on their back or more frequently on the left side with knees slightly bent. The shoulders and pelvis were usually leaned on the north side of the grave wall. Burial inventory consists of ceramic, bronze weapons, personal adornments and few domestic animal bones.

The Sabinka 2 site is archaeologically dated back to 1300-1100 BC [[27]](https://paperpile.com/c/jcKi2X/ovx3). There are a minimum of 60 mostly single or few double ground Karasuk burials and 28 were made in kurgans. Except for Karasuk graves 20, kurgans with early Iron Age Tes culture collective burials were also excavated. The SP75 individual was an adult male of about 30 years old and was found in a single grave of the kurgan 7. The maxilla of the individual is damaged in the area of the nasal sinuses. According to observations of A.Zubova, the individual has porotic lesions at sinus flour, which can be a result of chronic sinusitis. There are no other visible pathologies on the skull and bones.

**The Vác samples:**

During renovation of the White Monks’ church of Vác, Hungary in 1994 and 1995, two crypts were uncovered [[28,29]](https://paperpile.com/c/jcKi2X/cUIQ+aAB9). The coffins had been left undisturbed for almost two hundred years and contained the remains of 265 individuals of which six are included in this study (Table S1). Those buried in the crypts were conserved naturally. Mummification was made possible by the unique microclimate and the method of interment. The average temperature of the crypt was low, 8-11^o^ C (46.4 – 51.8 ^o^ F), independent of seasons and external temperature fluctuations. One critical determining factor for the mummification of these individuals was the slight but constant airflow through the two narrow air funnels connecting the undercroft to the outside world. Contributing to the mummification was also the fact that the deceased were placed in coffins made mostly of pine planks and pine wood shavings were placed under the body. The terpenoid content of pine prevented the growth of fungi and bacteria. As a result, the corpses dried up slowly instead of naturally decomposing [[30-39]](https://paperpile.com/c/jcKi2X/h4f3J+dOehr+Vkksm+5NAql+2UZzc+SjDgT+Bw7DO+xegXc+WjODU+LP7V7).

Based on the descriptions on the coffins and in the parish registers of death and baptisms, the crypt served as a burial place for people living in Vác between 1674 and 1838. The first burial took place in 1731. The archives and inscriptions on the coffins enabled many individuals to be identified by name and occupation – 166 individuals from the 265 are known by name.

The human remains from the crypts are housed in the Department of Anthropology of the Hungarian Natural History Museum, Budapest.

# Supplemental Tables:

Table S1. Samples used in this paper with basic information.

| Sample Name | Bone part | Location | Date ([BCE/CE cal 14C*] or [estimated^]) | Genetic sex | USER treated shotgun seq coverage (no methyl treatment) | ng/uL conc. of extract |
| --- | --- | --- | --- | --- | --- | --- |
| SP75 | cochlea | East Altai Mountains, Russia | 1191-1010 BCE* | M | 28.5 | 1.79 |
| Zvej16 | cochlea | Zvejnieki, Latvia | 5462-5220 BCE* | M | 28.9 | 1.83 |
| Vác 179 Petrous (HNHM Inv. No 2009.19.179.) | cochlea | Vác, Hungary | 1800s ^ | F | NA | 0.856 |
| Vác 179 Molar (HNHM Inv. No 2009.19.179.) | First molar | Vác, Hungary | 1800s ^ | F | NA | 0.33 |
| Vác 207 Petrous (HNHM Inv. No 2009.19.207.) | cochlea | Vác, Hungary | 1800s ^ | M | NA | Too low |
| Vác 207 Molar (HNHM Inv. No 2009.19.207.) | First molar | Vác, Hungary | 1800s ^ | M | NA | 0.224 |
| Vác 193 Petrous (HNHM Inv. No 2009.19.193.) | cochlea | Vác, Hungary | 1800s ^ | F | NA | Too low |
| Vác 193 Molar (HNHM Inv. No 2009.19.193.) | Second molar | Vác, Hungary | 1800s ^ | F | NA | 0.186 |
| Vác 164 Petrous (HNHM Inv. No 2009.19.164.) | cochlea | Vác, Hungary | 1800s ^ | F | NA | Too low |
| Vác 164 Molar (HNHM Inv. No 2009.19.164.) | First molar | Vác, Hungary | 1800s ^ | F | NA | Too low |
| Vác 77 Petrous (HNHM Inv. No 2009.19.77.) | cochlea | Vác, Hungary | 1800s ^ | F | NA | 0.74 |
| Vác 77 Molar (HNHM Inv. No 2009.19.77.) | First molar | Vác, Hungary | 1800s ^ | F | NA | 0.296 |
| Vác 210 Petrous (HNHM Inv. No 2009.19.210.) | cochlea | Vác, Hungary | 1800s ^ | F | NA | 0.736 |
| Vác 210 Molar (HNHM Inv. No 2009.19.210.) | First molar | Vác, Hungary | 1800s ^ | F | NA | 0.304 |
| 10658 | cochlea | Klosterneuburg, Austria | 26-407 CE* | F | 0.8 | 1.06 |
| 3547 | cochlea | Novo Selo, Croatia | 545-597 CE* | F | 0.9 | 0.824 |
| 3543 | cochlea | Gardun, Croatia | 431-600 CE* | M | 0.9 | 0.342 |
| 3544 | cochlea | Gardun, Croatia | 549-600 CE* | M | 1.0 | Too low |
| 3545 | cochlea | Gardun, Croatia | 431-542 CE* | F | 0.8 | 0.33 |
| 2050 | cochlea | Omišalj-Mirine, Croatia | 402-533 CE* | F | 0.9 | 1.05 |
| 11112 | cochlea | Isola Sacra, Italy | 1 -400 CE* | M | 1.1 | Too low |
| 11118 | cochlea | Isola Sacra, Italy | 1 -400 CE* | F | 1.7 | 1.29 |

Extract concentrations were measured using a Qubit3 high sensitivity double stranded DNA kit. Samples labeled as “Too low” were below the measurable threshold (0.1ng/uL).

Table S2. Samples with various treatments and sequencing strategies

| Sample Name | NEB + EMseq +/- exoVII treatment | dslib + EMseq +/- exoVII treatment | EMseq + sslib +/- exoVII treatment | Bisulfite treatment + sslib | Low-coverage shotgun seq (~5 million reads) | Increased shotgun sequencing (~30-60 million reads) | Twist methylome capture |
| --- | --- | --- | --- | --- | --- | --- | --- |
| SP75 | yes | yes | yes | yes | yes | yes | yes |
| Zvej16 | yes | yes | yes | yes | yes | yes | yes |
| Vác 179 Petrous | no | no | yes | yes | yes | no | no |
| Vác 179 Molar | no | no | yes | yes | yes | no | no |
| Vác 207 Petrous | no | no | yes | yes | yes | no | no |
| Vác 207 Molar | no | no | yes | yes | yes | no | no |
| Vác 193 Petrous | no | no | yes | yes | yes | no | no |
| Vác 193 Molar | no | no | yes | yes | yes | no | no |
| Vác 164 Petrous | no | no | yes | yes | yes | no | no |
| Vác 164 Molar | no | no | yes | yes | yes | no | no |
| Vác 77 Petrous | no | no | yes | yes | yes | no | no |
| Vác 77 Molar | no | no | yes | yes | yes | no | no |
| Vác 210 Petrous | no | no | yes | yes | yes | no | no |
| Vác 210 Molar | no | no | yes | yes | yes | no | no |
| 10658 | no | no | yes | yes | yes | no | no |
| 3547 | no | no | yes | yes | yes | no | no |
| 3543 | no | no | yes | yes | yes | no | no |
| 3544 | no | no | yes | yes | yes | no | no |
| 3545 | no | no | yes | yes | yes | no | no |
| 2050 | no | no | yes | yes | yes | no | no |
| 11112 | no | no | yes | yes | yes | no | no |
| 11118 | no | no | yes | yes | yes | no | no |

Table S3. List of oligonucleotides designed for this study

| Oligonucleotide Name | Sequence 5’ - 3’ | Description |
| --- | --- | --- |
| exovii-1 | TCGTCGTTTGGTATGGCTTCATTCAGCTCCGGTTCCCAACGATCAAGGCGAGTTACATGA | Test exonuclease VII cutting ability, strand with overhangs |
| exovii-2 | CGCCTTGATCGTTGGGAACCGGAGCTGAATGAAGCCATAC | Test exonuclease VII cutting ability, strand without overhangs |
| exovii-3 | T**U**GTCGT**U**TGGTATGGCTTCATTCAGCTCCGGTTCCCAACGATCAAGGCGA**U**TTA**U**ATGA | Test exonuclease VII cutting ability including Us, strand with overhangs |
| exovii-4 | T**mC**GTCGTT**mC**GGTATGGCTTCATTCAGCTCCGGTTCCCAACGATCAAGG**mC**GAGTTA**mC**ATGA | Test exonuclease VII cutting ability including mCs, strand with overhangs |
| full-methyl-pos-1 | TmCGTmCGTTTAGTATmCGmCGTmCGTTmCGAmCTTmCGATTmCGmCAAmCGATmCGmCGAmCGAGTTAmCATGA | Positive control containing 15 Cs, all of which are methylated, strand 1 |
| full-methyl-pos-2 | TmCATGTAAmCTmCGTmCGmCGATmCGTTGmCGAATmCGAAGTmCGAAmCGAmCGmCGATAmCTAAAmCGAmCGA | Positive control containing 15 Cs, all of which are methylated, strand 2 |
| no-methyl-pos-1 | TCGTCGTTTGGTATGGCTTCATTCAGCTCCGGTTCCCAACGATCAAGGCGAGTTACATGA | Positive control containing 15 Cs, none of which are methylated, strand 1 |
| no-methyl-pos-2 | TCATGTAACTCGCCTTGATCGTTGGGAACCGGAGCTGAATGAAGCCATACCAAACGACGA | Positive control containing 15 Cs, none of which are methylated, strand 2 |

Table S4. Percent endogenous.

|  | **sslib (no methyl conversion)** | **NEB + EMseq** | **exoVII + NEB + EMseq** | **ds-lib + EMseq** | **exoVII + ds-lib + EMseq** | **ss-lib + EMseq** | **exoVII + sslib+ EMseq** | **bisulfite + sslib** |
| --- | --- | --- | --- | --- | --- | --- | --- | --- |
| **Zvej16** | 53.6 | 39.1 | 15.7 | 21.5 | 16.6 | 46.6 | 29.1 | 52.5 |
| **SP75** | 70 | 59.4 | 7.6 | 29 | 22.8 | 47.3 | 45.4 | 66 |
| **Zvej16 (100pg input)** | 50.9 | 5.5 | 1.5 | 0.3 | 0.2 | 2.2 | 0.9 | 11.4 |
| **SP75 (100pg input)** | 58.3 | 7.9 | 3.7 | 1.1 | 1 | 4.1 | 2.2 | 14.8 |
| **Vác 179 Petrous** | 54.40 | NA | NA | NA | NA | 38.6 | 38.4 | 51.67 |
| **Vác 179 Molar** | 58.15 | NA | NA | NA | NA | 23.0 | 19.2 | 49.22 |
| **Vác 207 Petrous** | 21.47 | NA | NA | NA | NA | 7.8 | 5.5 | 19.74 |
| **Vác 207 Molar** | 2.29 | NA | NA | NA | NA | 0.2 | 0.2 | 1.91 |
| **Vác 193 Petrous** | 11.70 | NA | NA | NA | NA | 2.4 | 1.8 | 10.51 |
| **Vác 193 Molar** | 5.04 | NA | NA | NA | NA | 1.2 | 0.4 | 4.56 |
| **Vác 164 Petrous** | 1.69 | NA | NA | NA | NA | 0.6 | 0.3 | 1.05 |
| **Vác 164 Molar** | 23.77 | NA | NA | NA | NA | 2.1 | 0.8 | 14.99 |
| **Vác 77 Petrous** | 57.48 | NA | NA | NA | NA | 38.5 | 36.2 | 53.66 |
| **Vác 77 Molar** | 1.56 | NA | NA | NA | NA | 0.5 | 0.6 | 1.57 |
| **Vác 210 Petrous** | 14.02 | NA | NA | NA | NA | 3.4 | 2.3 | 9.63 |
| **Vác 210 Molar** | 72.01 | NA | NA | NA | NA | 51.9 | 46.5 | 65.31 |
| **10658** | 30.78 | NA | NA | NA | NA | 9.9 | 6.5 | 18.90 |
| **3547** | 46.31 | NA | NA | NA | NA | 17.3 | 10.4 | 38.47 |
| **3543** | 53.13 | NA | NA | NA | NA | 48.3 | 37.8 | 64.40 |
| **3544** | 68.12 | NA | NA | NA | NA | 55.8 | 36.2 | 68.19 |
| **3545** | 66.36 | NA | NA | NA | NA | 44.4 | 33.3 | 60.88 |
| **2050** | 66.92 | NA | NA | NA | NA | 64.5 | 59.1 | 69.67 |
| **11112** | 28.00 | NA | NA | NA | NA | 17.6 | 12.7 | 42.68 |
| **11118** | 49.13 | NA | NA | NA | NA | 36.6 | 30.7 | 49.01 |

Percent endogenous is calculated by dividing the number of mapped reads with a mapping quality of 30 or greater by the raw reads times 100.

Table S5. Percentage of mC in non-CpG contexts.

|  | **NEB + EMseq** | **exoVII + NEB + EMseq** | **ds-lib + EMseq** | **exoVII + ds-lib + EMseq** | **ss-lib+EMseq** | **exoVII + sslib+ EMseq** | **bisulfite + sslib** |
| --- | --- | --- | --- | --- | --- | --- | --- |
| **Zvej16** | 8.9 | 2.3 | 5.6 | 9.5 | 6.9 | 1.3 | 0.8 |
| **SP75** | 10 | 3.4 | 7 | 12 | 4.9 | 0.8 | 0.7 |
| **Zvej16 (100pg input)** | 1.3 | 1 | 20.1 | 17.6 | 2 | 3.3 | 1.1 |
| **SP75 (100pg input)** | 1.1 | 1 | 16.8 | 14.3 | 1.1 | 1.8 | 1 |
| **Vác 179 Petrous** | NA | NA | NA | NA | 35.75 | 32.6 | 0.7 |
| **Vác 179 Molar** | NA | NA | NA | NA | 74.25 | 57.45 | 0.6 |
| **Vác 207 Petrous** | NA | NA | NA | NA | 34.65 | 29.9 | 1.15 |
| **Vác 207 Molar** | NA | NA | NA | NA | 56.45 | 44.85 | 3.55 |
| **Vác 193 Petrous** | NA | NA | NA | NA | 47.15 | 22.05 | 1.4 |
| **Vác 193 Molar** | NA | NA | NA | NA | 46.6 | 38.7 | 16.35 |
| **Vác 164 Petrous** | NA | NA | NA | NA | 20.8 | 23.5 | 9.8 |
| **Vác 164 Molar** | NA | NA | NA | NA | 37 | 22.1 | 2.3 |
| **Vác 77 Petrous** | NA | NA | NA | NA | 33.35 | 24.3 | 0.6 |
| **Vác 77 Molar** | NA | NA | NA | NA | 80.15 | 77.3 | 2 |
| **Vác 210 Petrous** | NA | NA | NA | NA | 56.7 | 41.95 | 1 |
| **Vác 210 Molar** | NA | NA | NA | NA | 48.9 | 41.35 | 0.5 |
| **10658** | NA | NA | NA | NA | 31.3 | 14.4 | 0.8 |
| **3547** | NA | NA | NA | NA | 26.7 | 7.35 | 0.65 |
| **3543** | NA | NA | NA | NA | 22.8 | 3.75 | 0.65 |
| **3544** | NA | NA | NA | NA | 6.7 | 1.7 | 0.85 |
| **3545** | NA | NA | NA | NA | 30.3 | 10.25 | 1 |
| **2050** | NA | NA | NA | NA | 30.2 | 17.65 | 0.8 |
| **11112** | NA | NA | NA | NA | 2.35 | 1.2 | 0.75 |
| **11118** | NA | NA | NA | NA | 57.9 | 35.05 | 1.15 |

Table S6. Percentage of mC in CpG context.

|  | **NEB + EMseq** | **exoVII + NEB + EMseq** | **ds-lib + EMseq** | **exoVII + ds-lib + EMseq** | **ss-lib+EMseq** | **exoVII + sslib+ EMseq** | **bisulfite + sslib** |
| --- | --- | --- | --- | --- | --- | --- | --- |
| **Zvej16** | 65.1 | 52.6 | 66.5 | 69.6 | 69.1 | 69.6 | 71.2 |
| **SP75** | 66.1 | 53.4 | 70.3 | 73.3 | 72.2 | 72.7 | 74.7 |
| **Zvej16 (100pg input)** | 56.3 | 58.7 | 68.7 | 71.8 | 57.6 | 59.9 | 67.6 |
| **SP75 (100pg input)** | 57.7 | 60 | 72.1 | 73.3 | 61 | 64 | 71.6 |
| **Vác 179 Petrous** | NA | NA | NA | NA | 57.2 | 79.8 | 73.9 |
| **Vác 179 Molar** | NA | NA | NA | NA | 82.6 | 85.3 | 75.8 |
| **Vác 207 Petrous** | NA | NA | NA | NA | 55.9 | 78 | 76.9 |
| **Vác 207 Molar** | NA | NA | NA | NA | 55.1 | 50.1 | 46 |
| **Vác 193 Petrous** | NA | NA | NA | NA | 64.8 | 74.7 | 72.4 |
| **Vác 193 Molar** | NA | NA | NA | NA | 64.1 | 59.7 | 68 |
| **Vác 164 Petrous** | NA | NA | NA | NA | 46.9 | 58.4 | 45.7 |
| **Vác 164 Molar** | NA | NA | NA | NA | 55.6 | 48.1 | 58.6 |
| **Vác 77 Petrous** | NA | NA | NA | NA | 55.8 | 78.1 | 75.6 |
| **Vác 77 Molar** | NA | NA | NA | NA | 79.9 | 82.5 | 58.5 |
| **Vác 210 Petrous** | NA | NA | NA | NA | 71.6 | 75.2 | 74.8 |
| **Vác 210 Molar** | NA | NA | NA | NA | 70 | 82.5 | 76.2 |
| **10658** | NA | NA | NA | NA | 49 | 72.8 | 71.8 |
| **3547** | NA | NA | NA | NA | 47.3 | 68.9 | 69 |
| **3543** | NA | NA | NA | NA | 49.9 | 64.9 | 69.4 |
| **3544** | NA | NA | NA | NA | 41.6 | 74.8 | 72 |
| **3545** | NA | NA | NA | NA | 53.8 | 75.6 | 74.3 |
| **2050** | NA | NA | NA | NA | 52.2 | 74.9 | 72.7 |
| **11112** | NA | NA | NA | NA | 30.2 | 71 | 66.4 |
| **11118** | NA | NA | NA | NA | 71.3 | 79.2 | 75.5 |

Table S7. CpG island beta values of chromosome 1 across treatments.

| Treatment | Zvej16 | | SP75 | | Bone1 | | Bone 2 | |
| --- | --- | --- | --- | --- | --- | --- | --- | --- |
|  | mCs within CGI | mCs outside CGI | mCs within CGI | mCs outside CGI | mCs within CGI | mCs outside CGI | mCs within CGI | mCs outside CGI |
| NEB+EMseq | 21.4 | 65.6 | 21.2 | 66.7 | NA | NA | NA | NA |
| exoVII+NEB+EMseq | 10.7 | 53.1 | 8.8 | 54.0 | NA | NA | NA | NA |
| dslib+EMseq | 17.0 | 66.8 | 20.1 | 70.6 | NA | NA | NA | NA |
| exoVII+dslib+EMseq | 18.0 | 70.0 | 20.9 | 73.7 | NA | NA | NA | NA |
| sslib+EMseq | 18.1 | 69.6 | 16.9 | 72.7 | NA | NA | NA | NA |
| exoVII+sslib+EMseq | 13.7 | 70.4 | 13.4 | 73.4 | NA | NA | NA | NA |
| bisulfite+sslib | 24.5 | 71.5 | 28.1 | 75.2 | 13.5 | 74.1 | 14.0 | 75.3 |

The mean beta value in CpG context within and outside of CpG islands for each treatment.

Table S8. Distribution of beta values per bin.

|  | **Bins of beta values** | | | | | | | | | |
| --- | --- | --- | --- | --- | --- | --- | --- | --- | --- | --- |
| Sample | **1-10** | **11-20** | **21-30** | **31-40** | **41-50** | **51-60** | **61-70** | **71-80** | **81-90** | **91-100** |
| Osteoblast | 0.21 | 0.12 | 0.05 | 0.03 | 0.03 | 0.04 | 0.06 | 0.25 | 0.21 | 0.00 |
| Zvej16 RoAM 28x | 0.19 | 0.14 | 0.04 | 0.03 | 0.02 | 0.03 | 0.05 | 0.28 | 0.21 | 0.00 |
| SP75 RoAM 28x | 0.18 | 0.14 | 0.05 | 0.02 | 0.03 | 0.04 | 0.06 | 0.27 | 0.23 | 0.00 |
| ZVvej16 DM 28x | 0.32 | 0.05 | 0.04 | 0.04 | 0.05 | 0.08 | 0.24 | 0.16 | 0.02 | 0.00 |
| SP75 DM 28x | 0.32 | 0.05 | 0.04 | 0.04 | 0.06 | 0.13 | 0.24 | 0.11 | 0.01 | 0.00 |
| Zvej16 BS 0.27x | 0.20 | 0.08 | 0.05 | 0.04 | 0.04 | 0.05 | 0.16 | 0.27 | 0.09 | 0.01 |
| SP75 BS 0.29x | 0.19 | 0.08 | 0.05 | 0.04 | 0.04 | 0.04 | 0.11 | 0.29 | 0.14 | 0.02 |
| Zvej16 BS Twist | 0.26 | 0.07 | 0.04 | 0.03 | 0.03 | 0.05 | 0.18 | 0.25 | 0.05 | 0.00 |
| SP75 BS Twist | 0.22 | 0.08 | 0.04 | 0.04 | 0.04 | 0.04 | 0.11 | 0.28 | 0.12 | 0.02 |
| Zvej16 DM 0.5x | 0.11 | 0.20 | 0.18 | 0.10 | 0.04 | 0.02 | 0.01 | 0.00 | 0.00 | 0.00 |
| Zvej16 DM 1x | 0.12 | 0.11 | 0.14 | 0.17 | 0.13 | 0.06 | 0.03 | 0.01 | 0.00 | 0.00 |
| Zvej16 DM 5x | 0.25 | 0.07 | 0.04 | 0.05 | 0.08 | 0.19 | 0.17 | 0.05 | 0.01 | 0.00 |
| SP75 DM 0.5x | 0.13 | 0.21 | 0.16 | 0.08 | 0.03 | 0.01 | 0.01 | 0.00 | 0.00 | 0.00 |
| SP75 DM 1x | 0.12 | 0.12 | 0.17 | 0.16 | 0.10 | 0.05 | 0.02 | 0.01 | 0.00 | 0.00 |
| SP75 DM 5x | 0.25 | 0.08 | 0.04 | 0.05 | 0.11 | 0.20 | 0.13 | 0.03 | 0.01 | 0.00 |
| Zvej16 EMseq 0.46x | 0.21 | 0.09 | 0.05 | 0.04 | 0.04 | 0.05 | 0.11 | 0.28 | 0.12 | 0.01 |
| SP75 EMseq 0.61x | 0.21 | 0.08 | 0.04 | 0.03 | 0.03 | 0.09 | 0.17 | 0.19 | 0.10 | 0.02 |
| Zvej16 EMseq twist | 0.23 | 0.07 | 0.05 | 0.04 | 0.04 | 0.04 | 0.08 | 0.22 | 0.21 | 0.01 |
| SP75 EMseq twist | 0.31 | 0.06 | 0.04 | 0.03 | 0.03 | 0.06 | 0.12 | 0.21 | 0.13 | 0.02 |
| Bone 1 | 0.26 | 0.07 | 0.02 | 0.03 | 0.03 | 0.05 | 0.08 | 0.20 | 0.24 | 0.02 |
| Bone 2 | 0.25 | 0.08 | 0.02 | 0.02 | 0.03 | 0.03 | 0.06 | 0.19 | 0.27 | 0.04 |

Beta values are binned from 1-100 in bins of 10. Note that beta values of zero are not included. Coverage for shotgun data is shown in the legend. DM = DamMet, BS = bisulfite. Data produced by DamMet and RoAM are using high coverage USER treated data produced of both Zvej16 and SP75 as part of the Allen Ancient Genome Diversity Project. BS and EMseq data of Zvej16 and SP75 were produced from extractions made for this study of the same cochlea as the Allen Ancient Genome Diversity Project and methyl treated either using bisulfite treatment or EMseq treatment in combination with single stranded library preparation. Twist captured data of the methylation treated libraries is also included. Bone 1 and Bone 2 are bisulfite treated samples.

Table S9. Substitution rate of C (in reference genome) to T (in sequenced read) at the 5’ read end of the high coverage Zvej16 data. The C to T rates in the beta bin columns are only measured in CpG context. The last column shows the C to T rate at all dinucleotide positions. As the high coverage data is only partially UDG treated, the very first position still shows some deamination signal.

| Position from 5’ end | beta bin below 10 | beta bin 10-20 | beta bin 20-30 | beta bin 30-40 | beta bin 40-50 | beta bin 50-60 | beta bin 60-70 | beta bin 70-80 | beta bin 80-90 | beta bin above 90 | C to T at all dinucleotide positions |
| --- | --- | --- | --- | --- | --- | --- | --- | --- | --- | --- | --- |
| 1 | 0.135 | 0.152 | 0.187 | 0.214 | 0.241 | 0.265 | 0.289 | 0.297 | 0.291 | 0.261 | 0.092 |
| 2 | 0.008 | 0.023 | 0.053 | 0.090 | 0.123 | 0.136 | 0.157 | 0.165 | 0.163 | 0.125 | 0.007 |
| 3 | 0.006 | 0.016 | 0.035 | 0.057 | 0.076 | 0.087 | 0.102 | 0.106 | 0.105 | 0.099 | 0.005 |
| 4 | 0.004 | 0.013 | 0.024 | 0.036 | 0.055 | 0.065 | 0.076 | 0.079 | 0.081 | 0.080 | 0.004 |
| 5 | 0.004 | 0.008 | 0.020 | 0.029 | 0.043 | 0.049 | 0.060 | 0.059 | 0.060 | 0.076 | 0.003 |
| 6 | 0.003 | 0.008 | 0.018 | 0.025 | 0.033 | 0.042 | 0.048 | 0.050 | 0.049 | 0.047 | 0.003 |
| 7 | 0.003 | 0.007 | 0.014 | 0.023 | 0.031 | 0.033 | 0.040 | 0.041 | 0.041 | 0.038 | 0.002 |
| 8 | 0.003 | 0.006 | 0.013 | 0.020 | 0.027 | 0.029 | 0.035 | 0.035 | 0.035 | 0.039 | 0.002 |
| 9 | 0.002 | 0.005 | 0.009 | 0.017 | 0.024 | 0.027 | 0.030 | 0.032 | 0.029 | 0.031 | 0.002 |
| 10 | 0.002 | 0.005 | 0.009 | 0.013 | 0.019 | 0.024 | 0.027 | 0.027 | 0.027 | 0.027 | 0.002 |
| 11 | 0.002 | 0.004 | 0.008 | 0.013 | 0.022 | 0.022 | 0.025 | 0.024 | 0.024 | 0.032 | 0.002 |
| 12 | 0.002 | 0.004 | 0.007 | 0.012 | 0.018 | 0.019 | 0.023 | 0.023 | 0.022 | 0.022 | 0.001 |
| 13 | 0.002 | 0.004 | 0.008 | 0.012 | 0.018 | 0.018 | 0.022 | 0.022 | 0.022 | 0.024 | 0.001 |
| 14 | 0.001 | 0.004 | 0.007 | 0.015 | 0.016 | 0.018 | 0.020 | 0.021 | 0.021 | 0.026 | 0.001 |
| 15 | 0.002 | 0.004 | 0.006 | 0.011 | 0.016 | 0.017 | 0.020 | 0.019 | 0.020 | 0.023 | 0.001 |
| 16 | 0.002 | 0.003 | 0.007 | 0.009 | 0.016 | 0.017 | 0.019 | 0.019 | 0.019 | 0.031 | 0.001 |
| 17 | 0.002 | 0.003 | 0.006 | 0.011 | 0.014 | 0.016 | 0.017 | 0.018 | 0.019 | 0.023 | 0.001 |
| 18 | 0.001 | 0.002 | 0.005 | 0.010 | 0.012 | 0.015 | 0.017 | 0.016 | 0.016 | 0.019 | 0.001 |
| 19 | 0.001 | 0.003 | 0.006 | 0.010 | 0.013 | 0.014 | 0.016 | 0.015 | 0.016 | 0.010 | 0.001 |
| 20 | 0.001 | 0.003 | 0.004 | 0.008 | 0.011 | 0.014 | 0.015 | 0.014 | 0.016 | 0.012 | 0.001 |

Table S10. Substitution rate of C (in reference genome) to T (in sequenced read) at the 5’ read end of the high coverage SP75 data. The C to T rates in the beta bin columns are only measured in CpG context. The last column shows the C to T rate at all dinucleotide positions. As the high coverage data is only partially UDG treated, the very first position still shows some deamination signal.

| Position from 5’ end | beta bin below 10 | beta bin 10-20 | beta bin 20-30 | beta bin 30-40 | beta bin 40-50 | beta bin 50-60 | beta bin 60-70 | beta bin 70-80 | beta bin 80-90 | beta bin above 90 | C to T at all dinucleotide positions |
| --- | --- | --- | --- | --- | --- | --- | --- | --- | --- | --- | --- |
| 1 | 0.105 | 0.112 | 0.129 | 0.151 | 0.179 | 0.221 | 0.241 | 0.247 | 0.251 | 0.253 | 0.075 |
| 2 | 0.006 | 0.015 | 0.028 | 0.049 | 0.071 | 0.103 | 0.126 | 0.134 | 0.136 | 0.135 | 0.006 |
| 3 | 0.004 | 0.010 | 0.021 | 0.031 | 0.047 | 0.068 | 0.080 | 0.083 | 0.083 | 0.075 | 0.004 |
| 4 | 0.003 | 0.007 | 0.016 | 0.022 | 0.036 | 0.049 | 0.062 | 0.063 | 0.066 | 0.066 | 0.003 |
| 5 | 0.003 | 0.006 | 0.011 | 0.017 | 0.028 | 0.039 | 0.048 | 0.050 | 0.051 | 0.050 | 0.002 |
| 6 | 0.003 | 0.004 | 0.009 | 0.014 | 0.023 | 0.034 | 0.041 | 0.042 | 0.043 | 0.040 | 0.002 |
| 7 | 0.002 | 0.004 | 0.009 | 0.012 | 0.022 | 0.028 | 0.035 | 0.036 | 0.036 | 0.033 | 0.002 |
| 8 | 0.002 | 0.004 | 0.007 | 0.012 | 0.019 | 0.026 | 0.030 | 0.030 | 0.030 | 0.032 | 0.002 |
| 9 | 0.002 | 0.003 | 0.006 | 0.012 | 0.018 | 0.023 | 0.026 | 0.026 | 0.026 | 0.023 | 0.002 |
| 10 | 0.002 | 0.003 | 0.007 | 0.010 | 0.013 | 0.020 | 0.024 | 0.023 | 0.023 | 0.023 | 0.002 |
| 11 | 0.001 | 0.003 | 0.005 | 0.009 | 0.015 | 0.020 | 0.022 | 0.021 | 0.021 | 0.020 | 0.001 |
| 12 | 0.001 | 0.002 | 0.005 | 0.009 | 0.014 | 0.018 | 0.020 | 0.019 | 0.020 | 0.017 | 0.002 |
| 13 | 0.001 | 0.002 | 0.004 | 0.008 | 0.015 | 0.016 | 0.019 | 0.018 | 0.018 | 0.017 | 0.001 |
| 14 | 0.001 | 0.002 | 0.005 | 0.008 | 0.011 | 0.015 | 0.018 | 0.017 | 0.016 | 0.015 | 0.001 |
| 15 | 0.001 | 0.002 | 0.005 | 0.007 | 0.012 | 0.013 | 0.017 | 0.016 | 0.016 | 0.015 | 0.001 |
| 16 | 0.001 | 0.002 | 0.005 | 0.006 | 0.009 | 0.013 | 0.016 | 0.015 | 0.015 | 0.011 | 0.001 |
| 17 | 0.001 | 0.002 | 0.005 | 0.006 | 0.012 | 0.012 | 0.015 | 0.014 | 0.015 | 0.013 | 0.001 |
| 18 | 0.001 | 0.002 | 0.004 | 0.007 | 0.010 | 0.011 | 0.015 | 0.013 | 0.014 | 0.015 | 0.001 |
| 19 | 0.001 | 0.002 | 0.004 | 0.004 | 0.011 | 0.011 | 0.014 | 0.013 | 0.013 | 0.011 | 0.001 |
| 20 | 0.001 | 0.002 | 0.004 | 0.006 | 0.009 | 0.011 | 0.013 | 0.012 | 0.012 | 0.010 | 0.001 |

# Supplemental Figures:


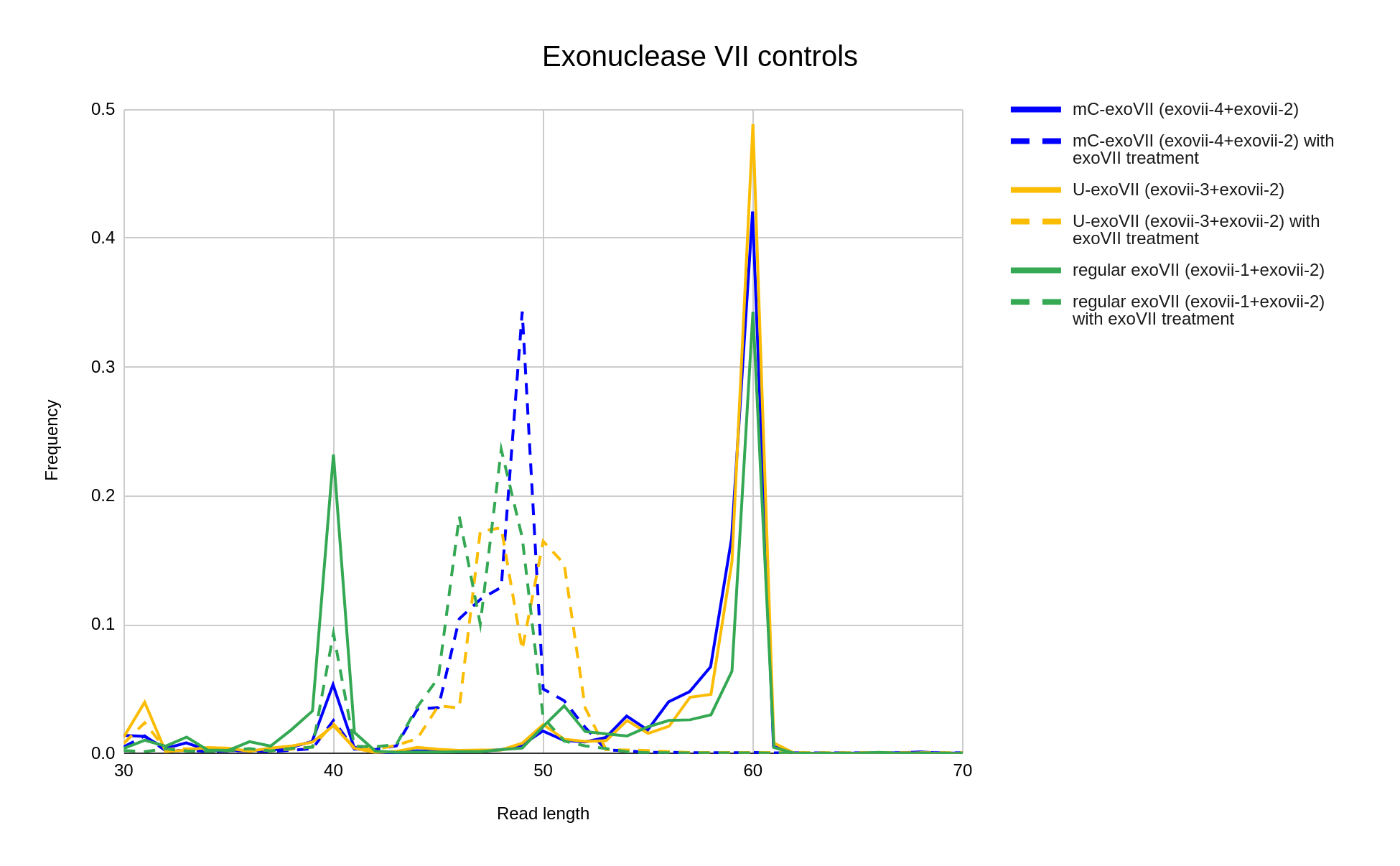


Figure S1. Exonuclease VII controls. In order to understand how well the exonuclease activity of exonuclease VII acts on overhangs, and what may affect the activity, we show the frequency of reads with various read lengths for each type of experiment.


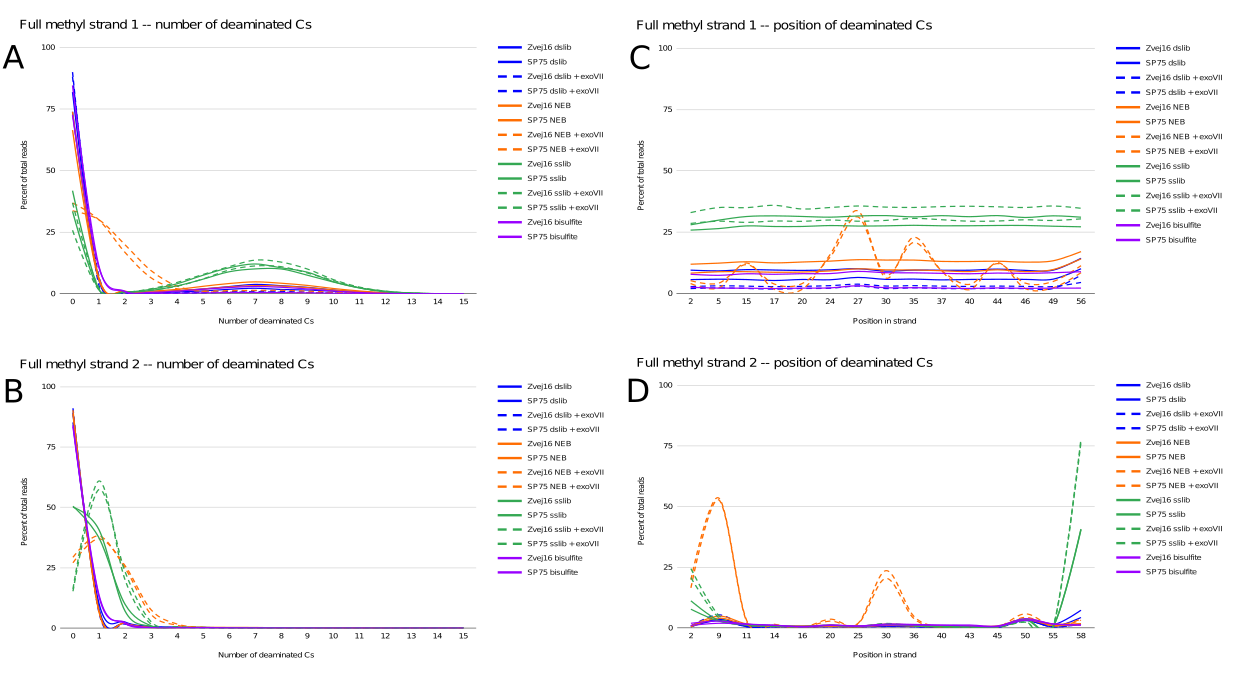


Figure S2. Full methyl positive controls. A) and B) depict the number of Cs that are deaminated in each of the two strands of the positive control. As this control has all 15 Cs in the 60bp control methylated, we expect none of the Cs to be deaminated if the positive control works with perfect efficiency. C) and D) depict the position in the 60bp strand that has a C and is deaminated.


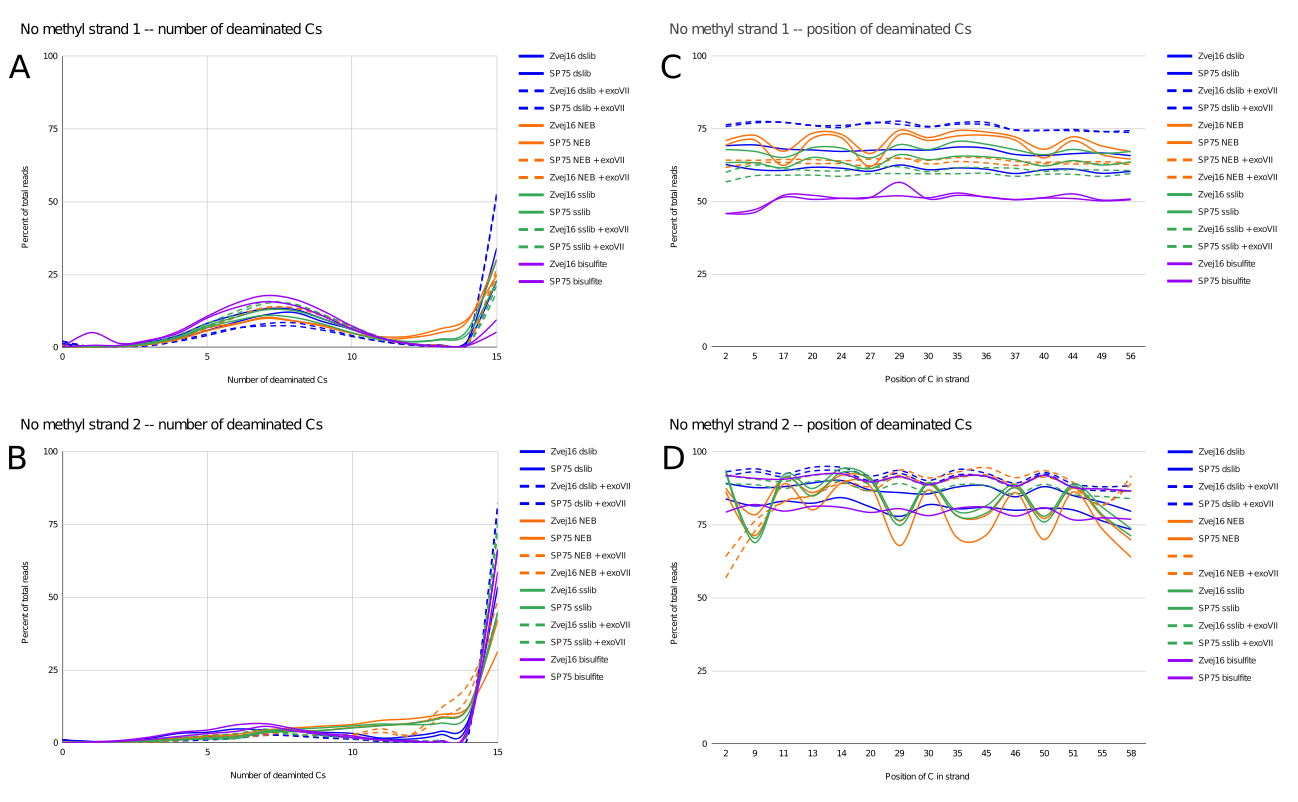


Figure S3. No methyl positive controls. A) and B) depict the number Cs that are deaminated in each of the two strands of the positive control. As this control has none of the 15 Cs in the 60bp control methylated, we expect all of the Cs to be deaminated if the positive control works with perfect efficiency. C) and D) depict the position in the 60bp strand that has a C and is deaminated.


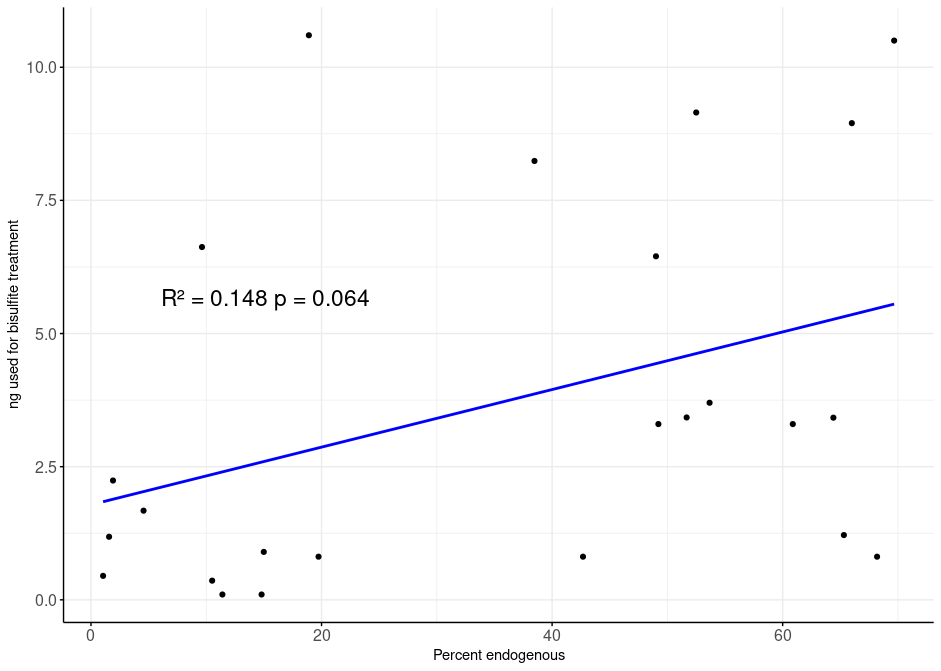


Figure S4. A scatterplot of percent endogenous versus the amount of ng used as input for bisulfite treatment. Each dot represents one of the samples from Table S4.


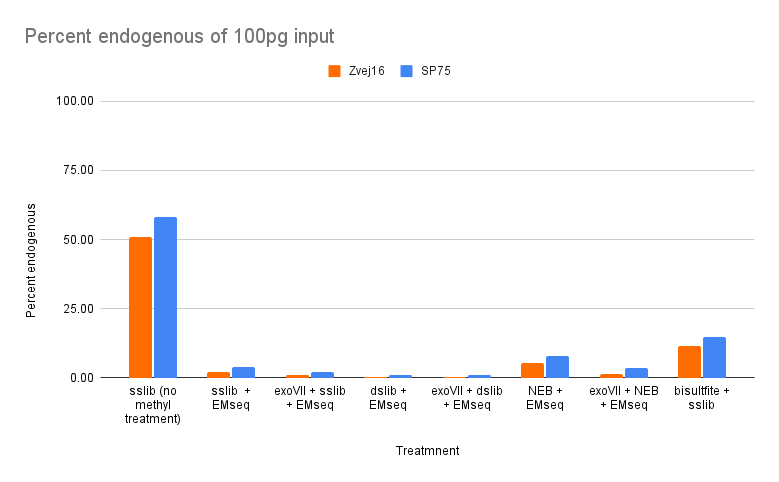


Figure S5. Percent endogenous of Zvej16 and SP75 of various treatments with 100pg input.


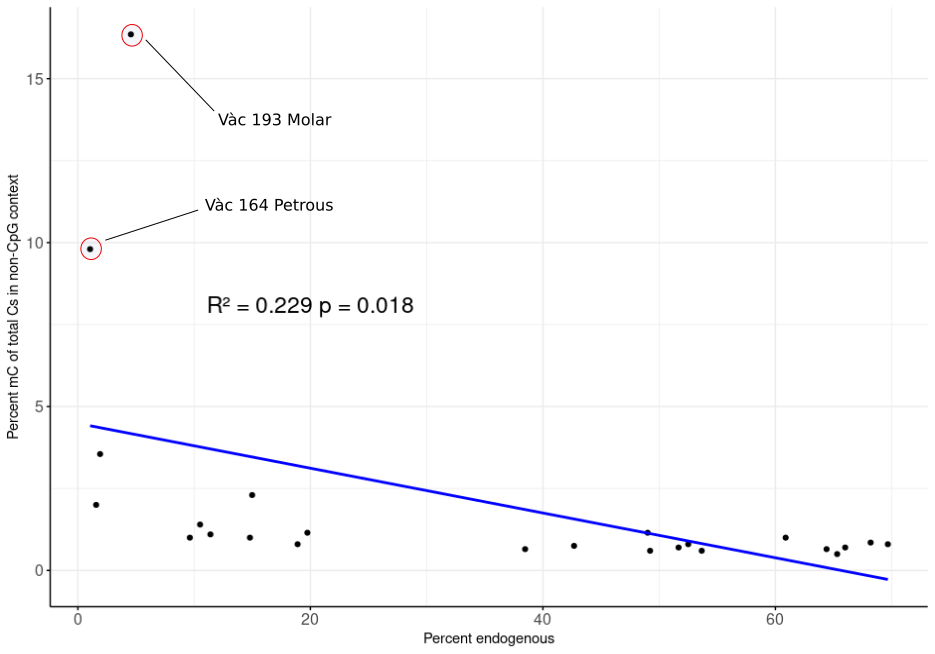


Figure S6. A scatterplot of percent endogenous versus the percentage of mCs of total Cs in a non-CpG context. Each dot represents one of the samples from Table S4. The two samples with the highest percentage of mCs in non-CpG context are shown.


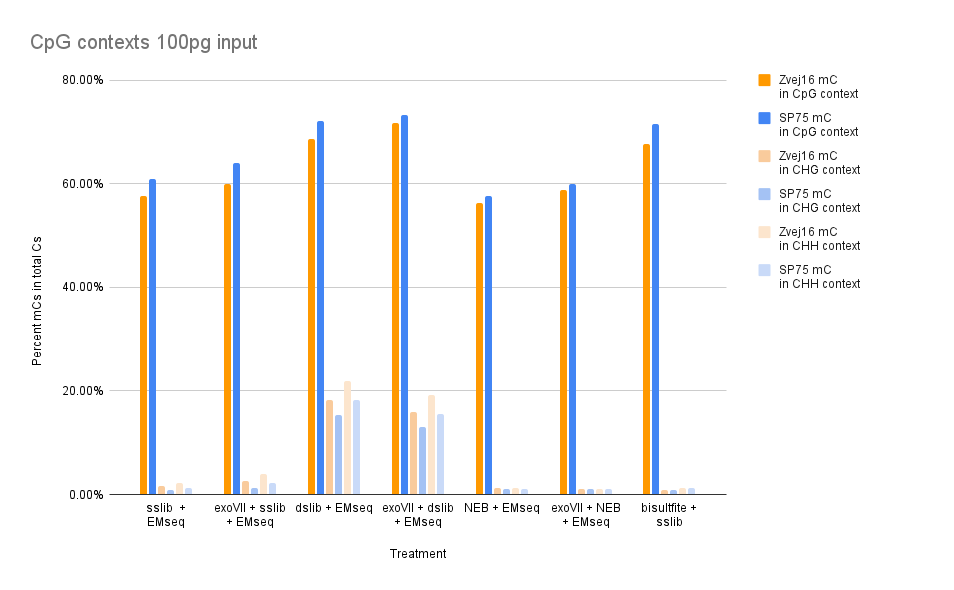


Figure S7. Percentage of mCs in different C-contexts of Zvej16 and SP75 of various treatments with 100pg input.


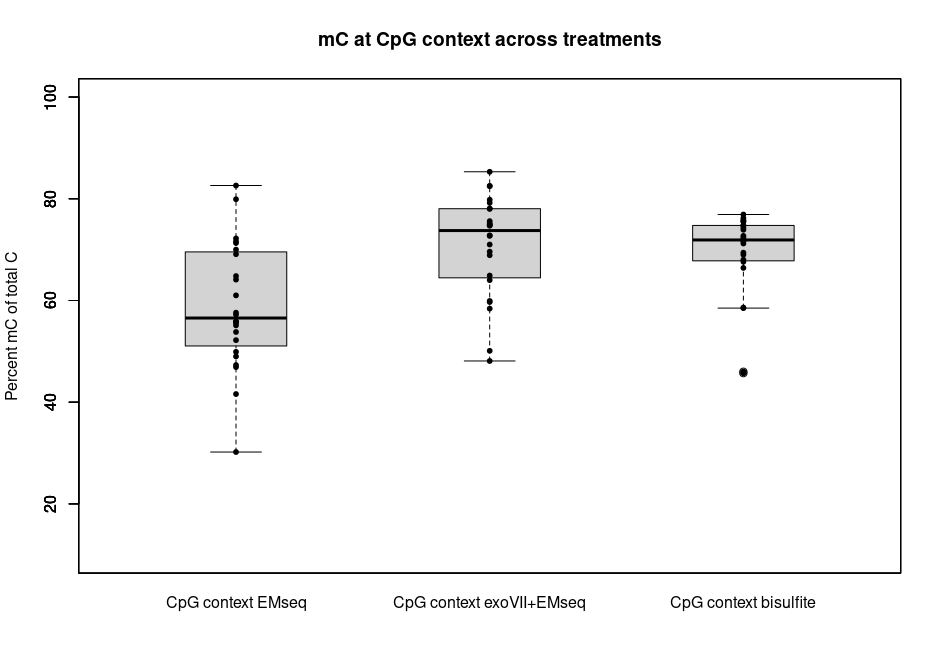


Figure S8. The percentage of methylated Cs of all Cs in CpG context. This is of all samples used in this study comparing the EMseq method with and without exonuclease VII treatment as well as bisulfite treatment in combination with the singles stranded library method. This comparison is restricted to CpG contexts.


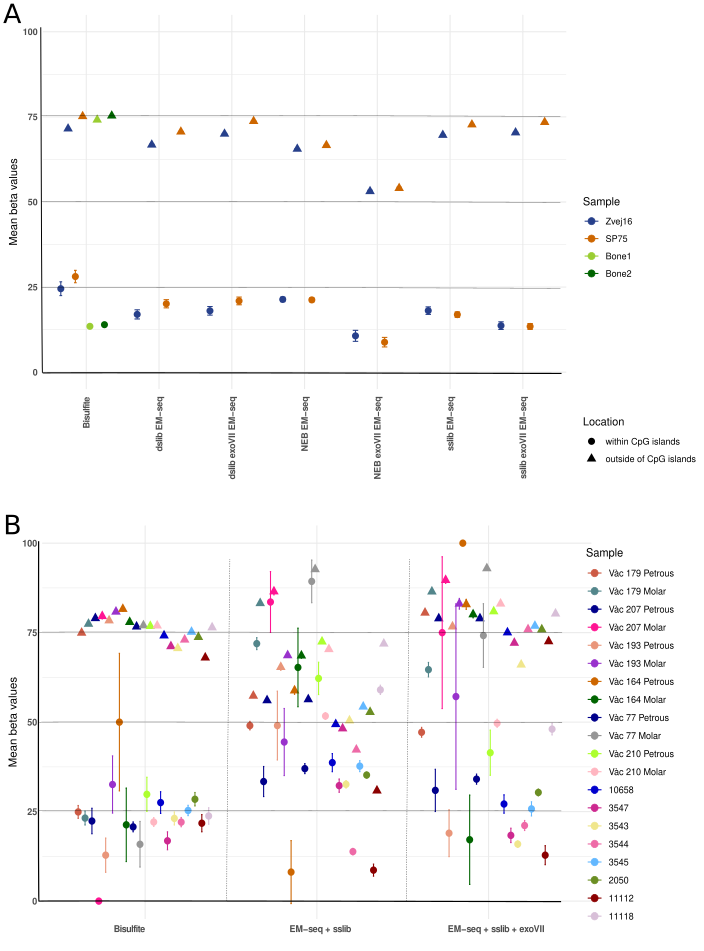


Figure S9. Mean beta values of chromosome 1 within and outside of CpG islands with 95% CI. A) Across treatments for Zvej16 and SP75 with Bone1 and Bone2 as comparison. B) Comparing bisulfite treatment with EMseq-sslib and EMseq-sslib_exoVII for additional 20 samples. Average number of reads used to calculate these rates is 920,000.


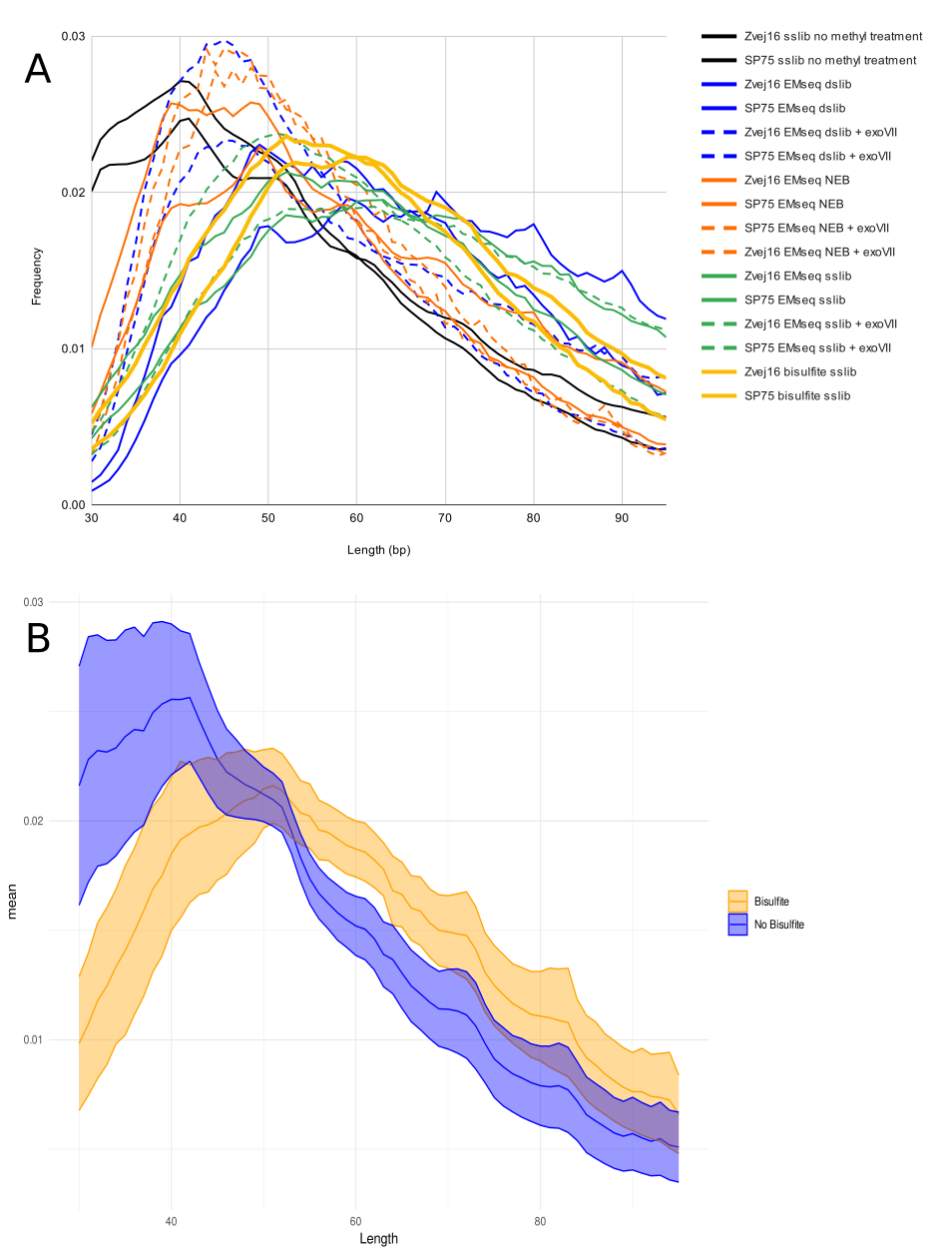


Figure S10. Length distributions. A) Length distributions of Zvej16 and SP75 after each of the different treatments. B) Length distributions of all samples from Table S2 comparing single stranded libraries that were pretreated with bisulfite treatment (Bisulfite) and single stranded libraries with no pretreatment of any kind (No Bisulfite).


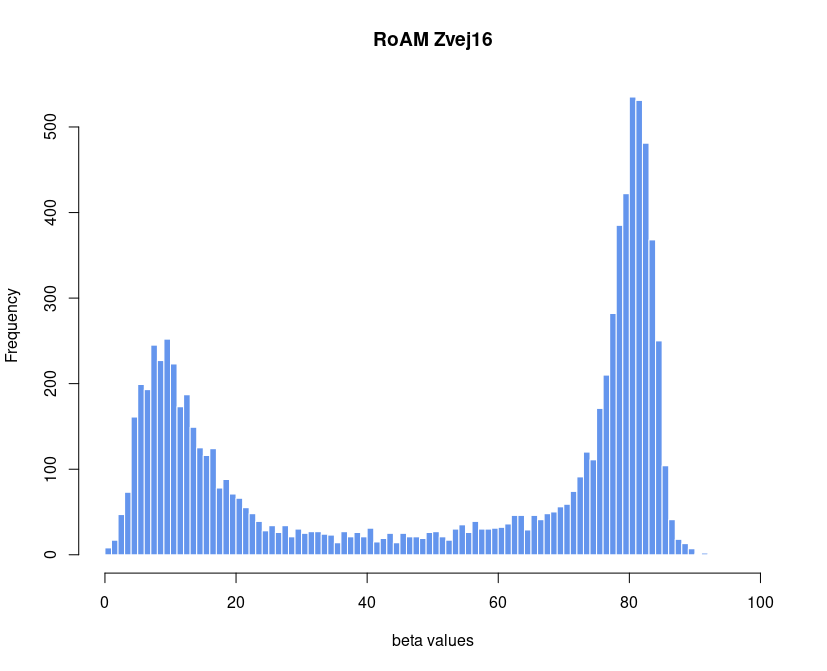
Figure S11. Histogram of segmented beta values of chromosome 1 of the 28x-fold data of Zvej16 after inferencing beta using RoAM.
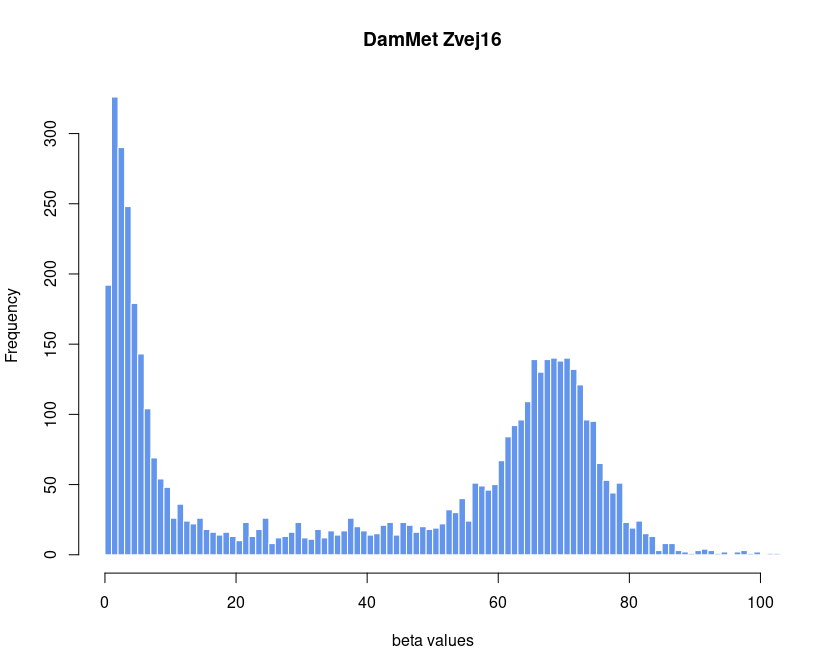
Figure S12. Histogram of segmented beta values of chromosome 1 of the 28x-fold data of Zvej16 after inferencing beta using DamMet.
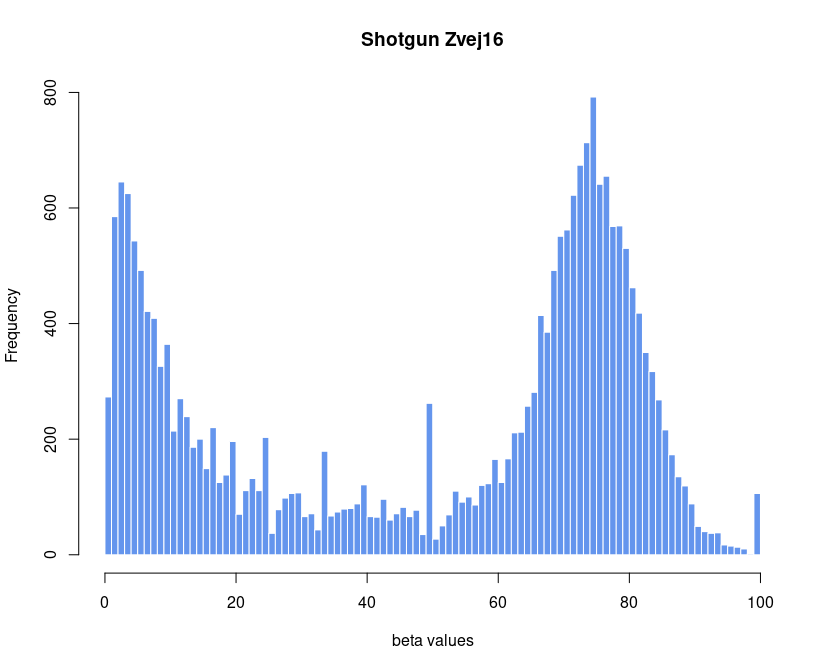
Figure S13. Histogram of segmented beta values of chromosome 1 of Zvej16 after bisulfite treatment. Beta values were calculated from shotgun data from 0.27-fold coverage.
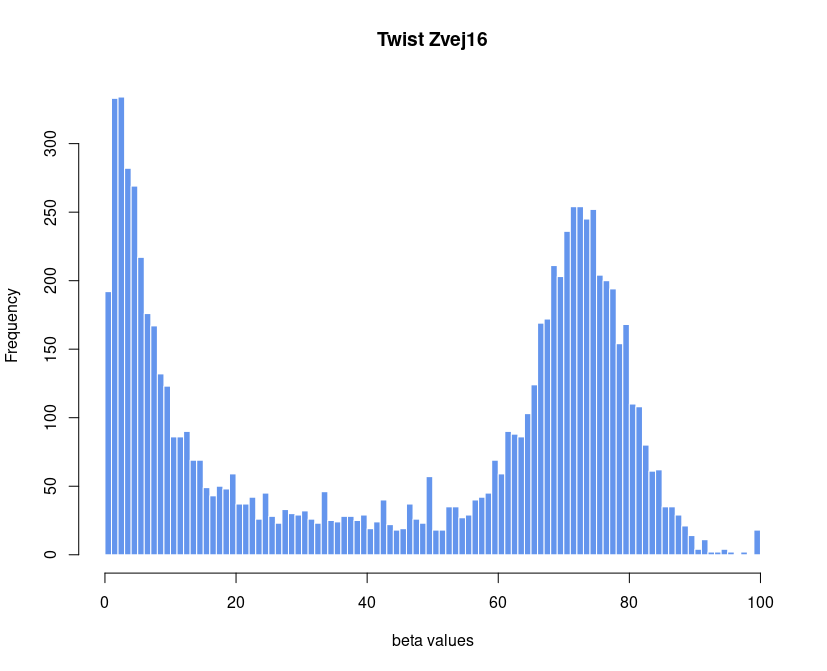
Figure S14. Histogram of segmented beta values of chromosome 1 of Zvej16 after bisulfite treatment and methylome capture using the Twist capture system.
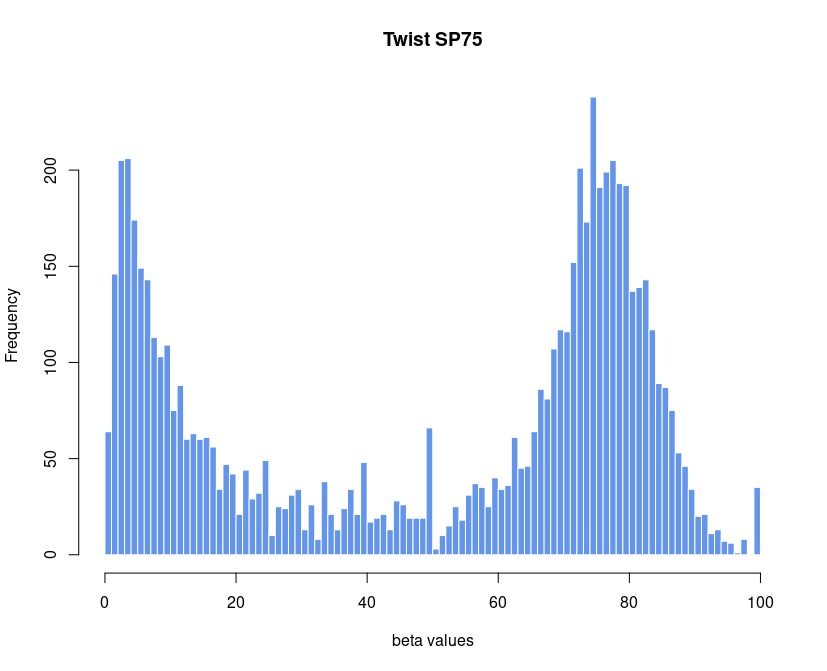
Figure S15. Histogram of segmented beta values of chromosome 1 of SP75 after bisulfite treatment and methylome capture using the Twist capture system.
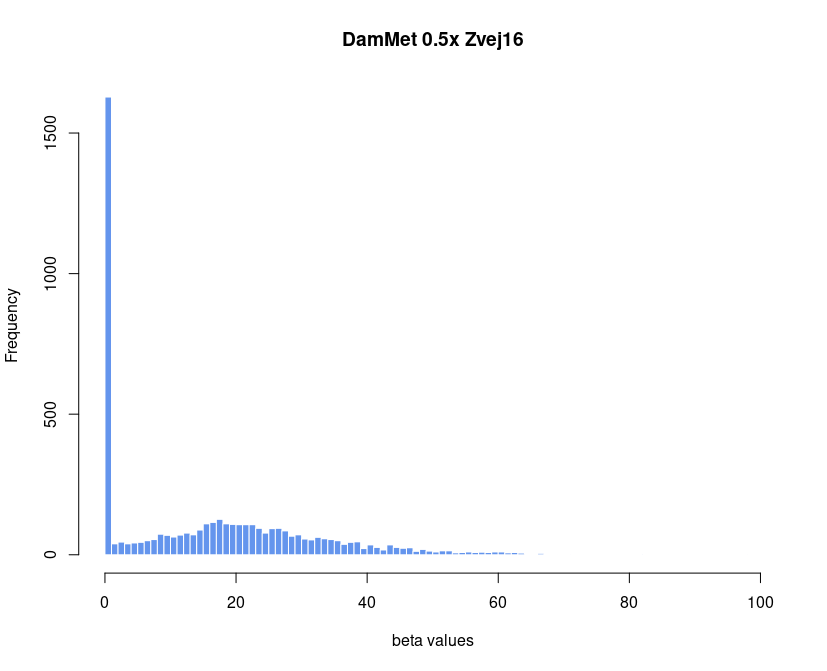
Figure S16. Histogram of segmented beta values of chromosome 1 of Zvej16 after downsampling the 28-fold non-methylation treated shotgun data to 0.5-fold coverage. Beta values were inferred using DamMet.
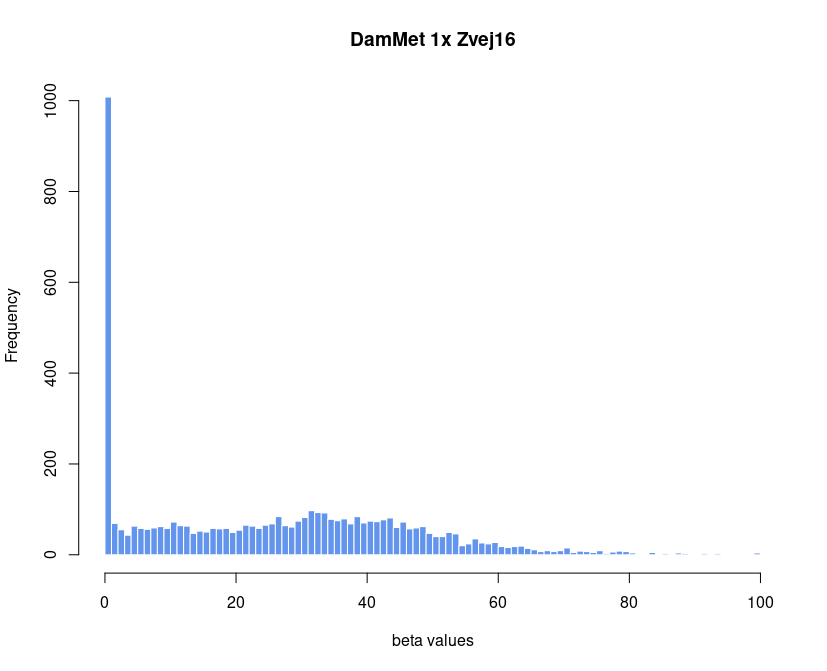
Figure S17. Histogram of segmented beta values of chromosome 1 of Zvej16 after downsampling the 28-fold non-methylation treated shotgun data to 1-fold coverage. Beta values were inferred using DamMet.
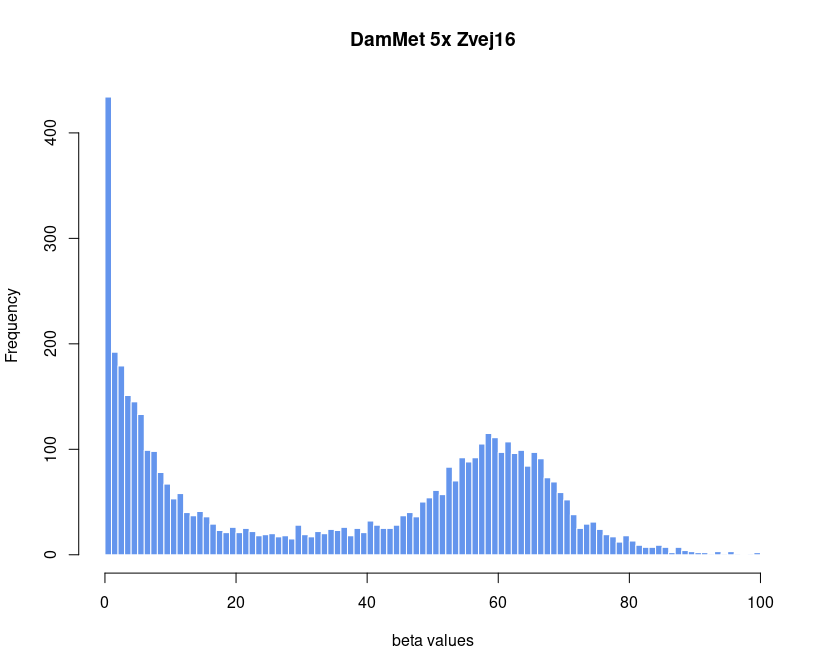
Figure S18. Histogram of segmented beta values of chromosome 1 of Zvej16 after downsampling the 28-fold non-methylation treated shotgun data to 5-fold coverage. Beta values were inferred using DamMet.
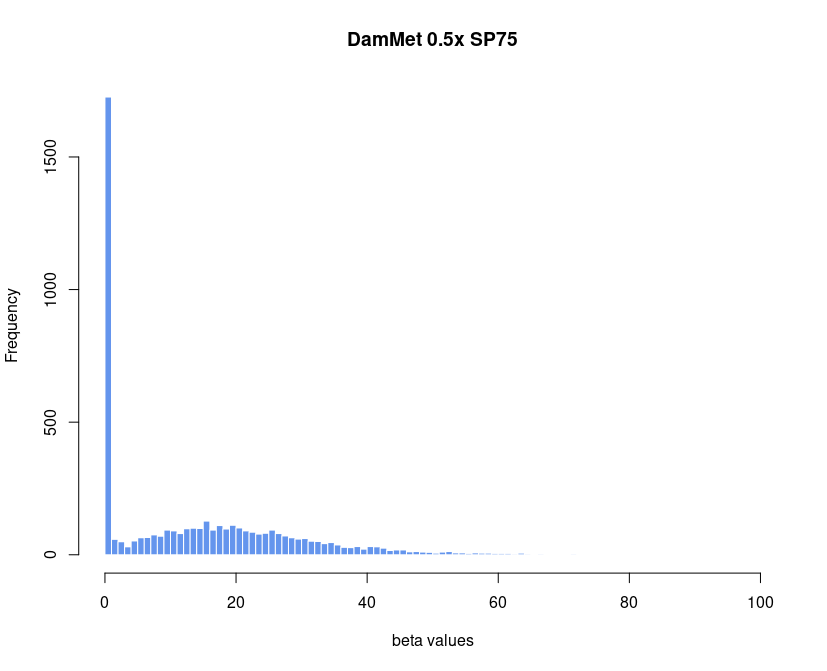
Figure S19. Histogram of segmented beta values of chromosome 1 of SP75 after downsampling the 28-fold non-methylation treated shotgun data to 0.5-fold coverage. Beta values were inferred using DamMet.
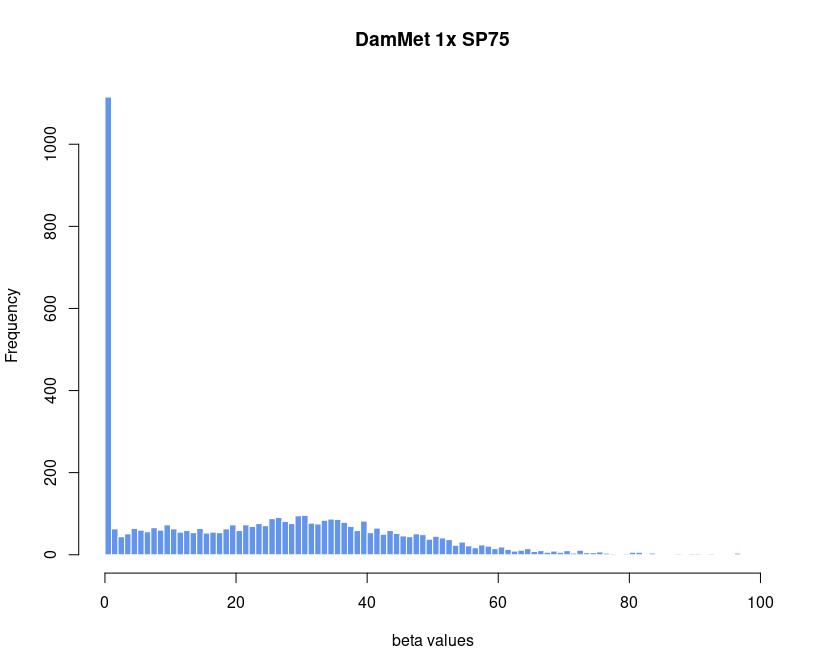
Figure S20. Histogram of segmented beta values of chromosome 1 of SP75 after downsampling the 28-fold non-methylation treated shotgun data to 1-fold coverage. Beta values were inferred using DamMet.
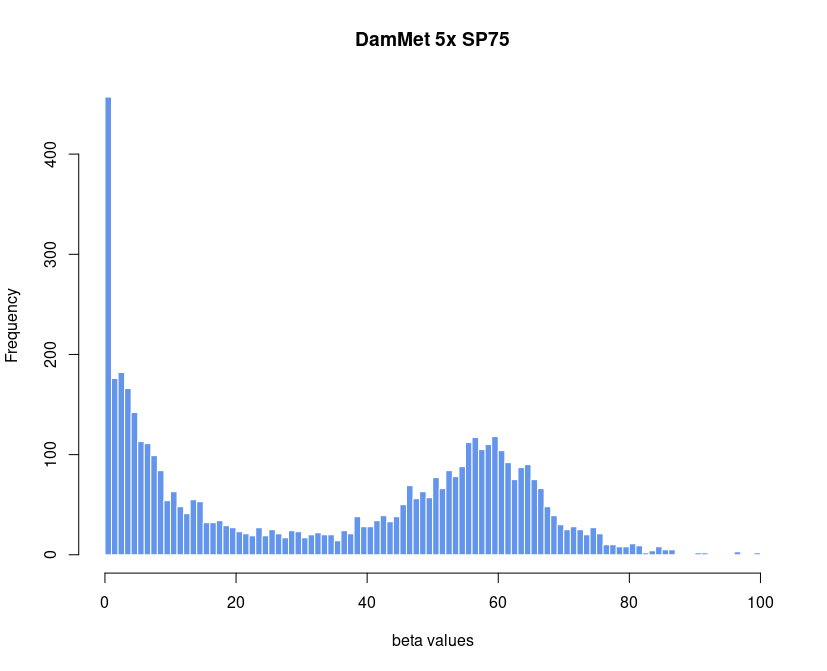


Figure S21. Histogram of segmented beta values of chromosome 1 of SP75 after downsampling the 28-fold non-methylation treated shotgun data to 5-fold coverage. Beta values were inferred using DamMet.


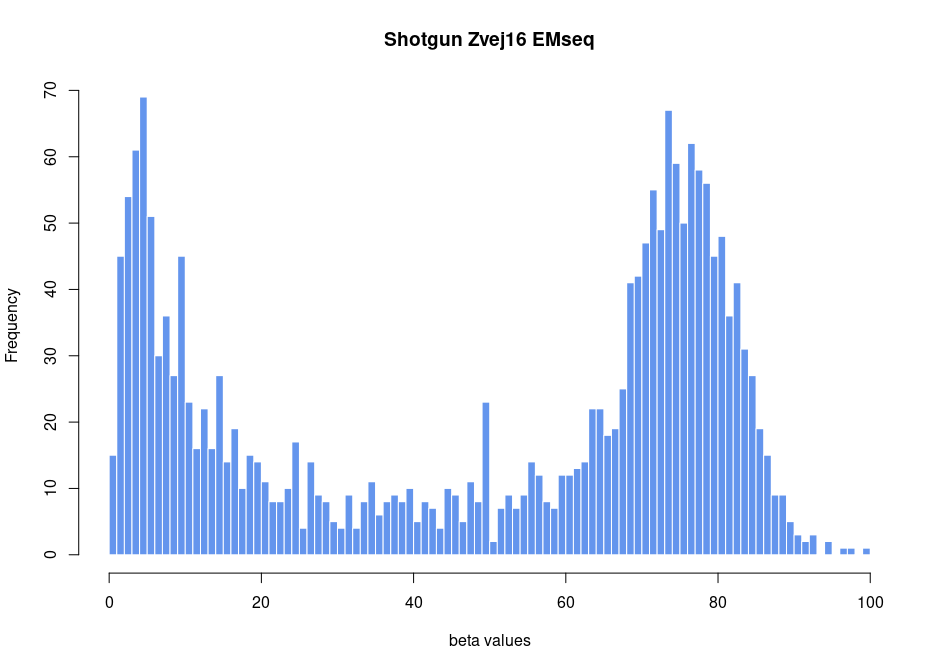
Figure S22. Histogram of segmented beta values of chromosome 1 of Zvej16 after EMseq treatment. The sample was shotgun sequenced to 0.46-fold coverage.
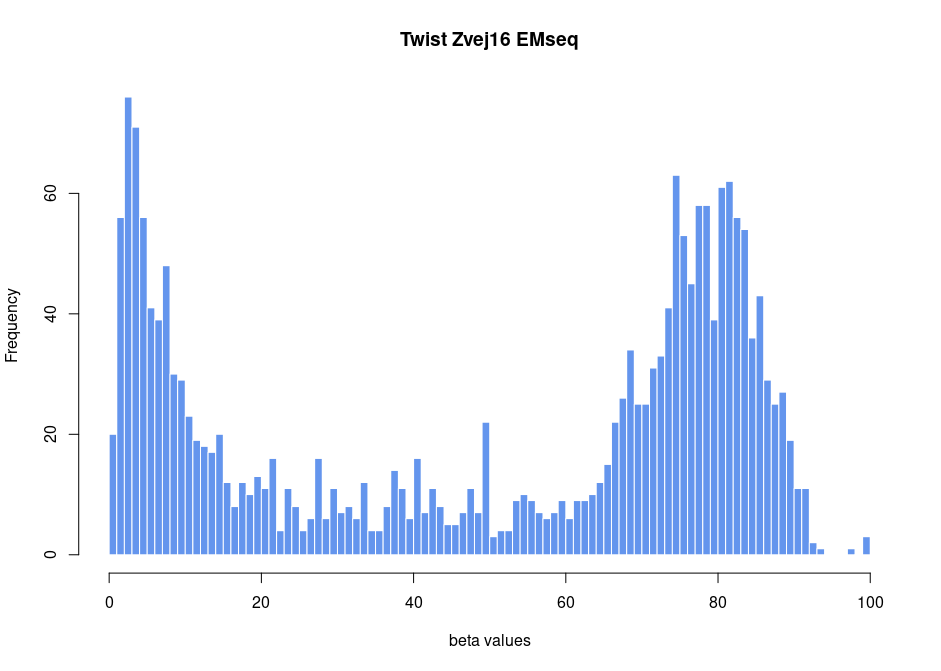
Figure S23. Histogram of segmented beta values of chromosome 1 of Zvej16 after EMseq treatment. The sample was sequenced after methylome capture using the Twist capture protocol.
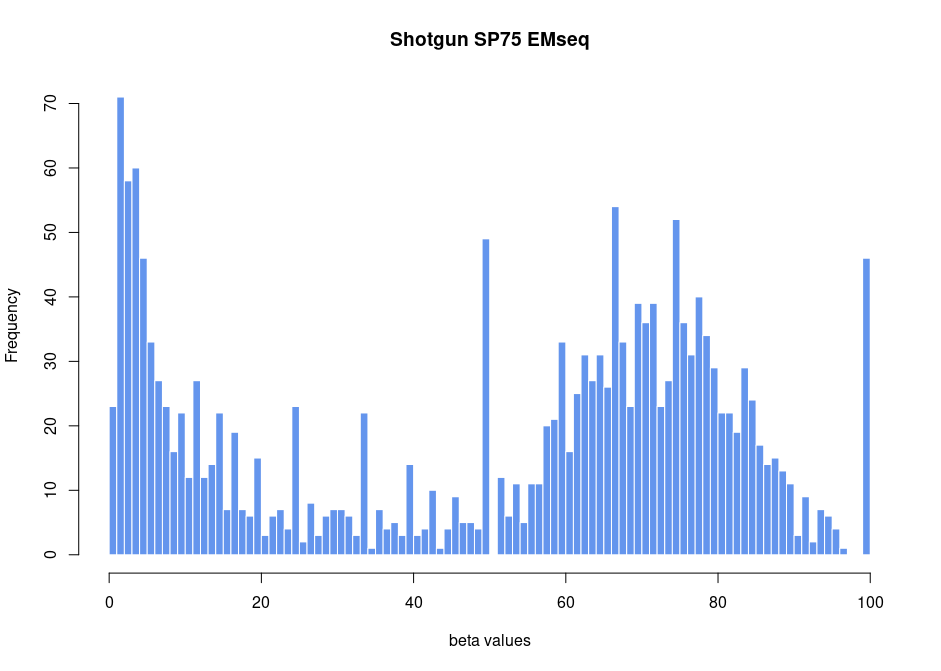
Figure S24. Histogram of segmented beta values of chromosome 1 of SP75 after EMseq treatment. The sample was shotgun sequenced to 0.61-fold coverage.


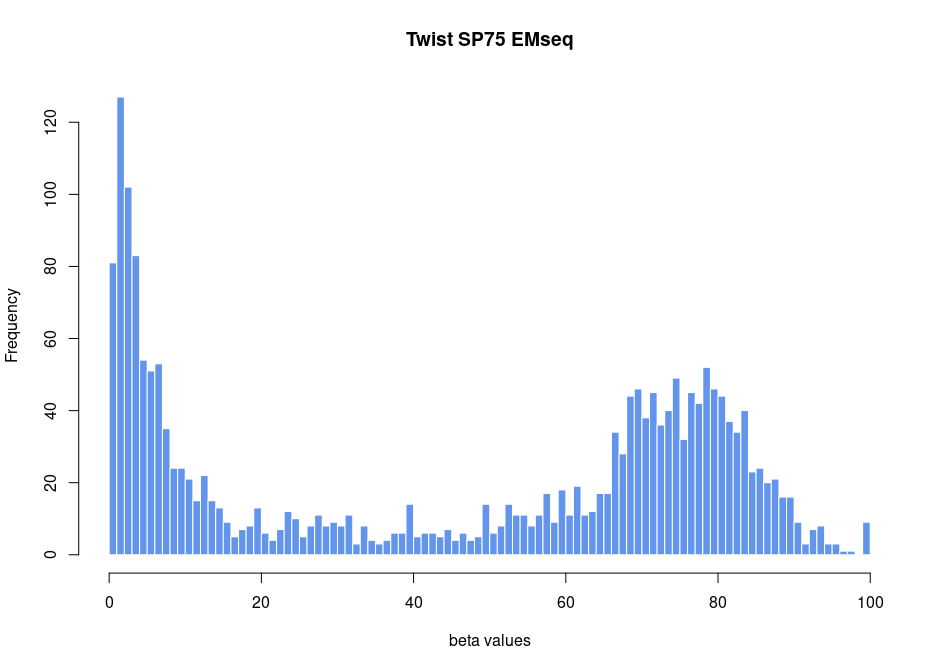


Figure S25. Histogram of segmented beta values of chromosome 1 of SP75 after EMseq treatment. The sample was sequenced after methylome capture using the Twist capture protocol.


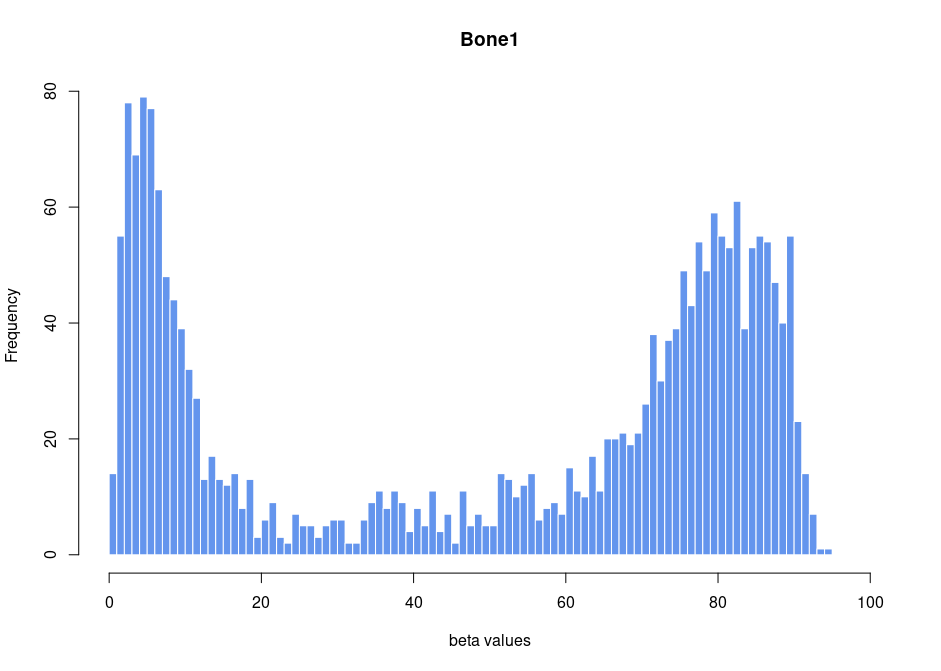


Figure S26. Histogram of segmented beta values of chromosome 1 of Bone 1.


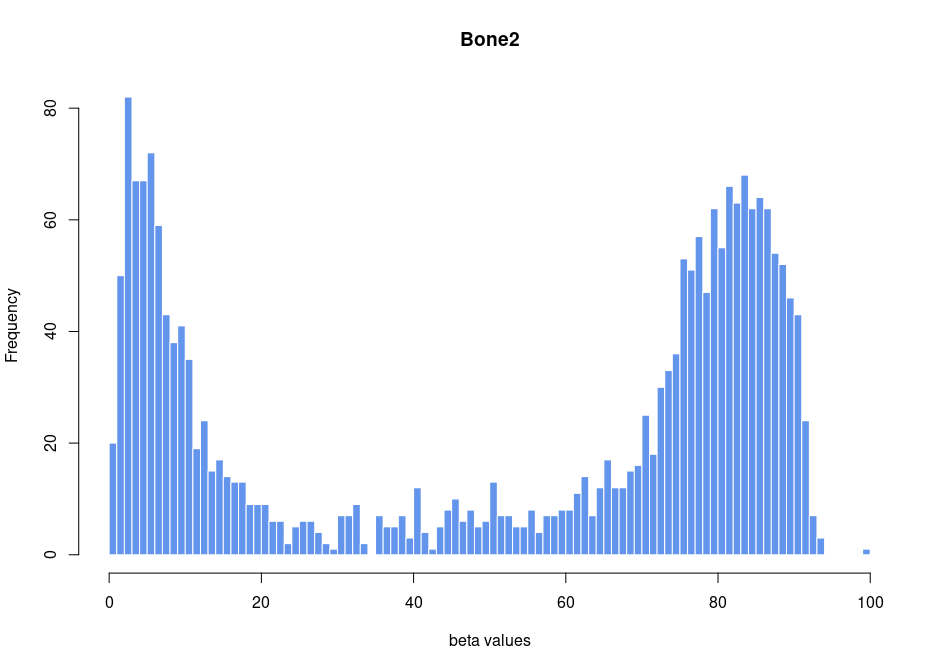


Figure S27. Histogram of segmented beta values of chromosome 1 of Bone 2.


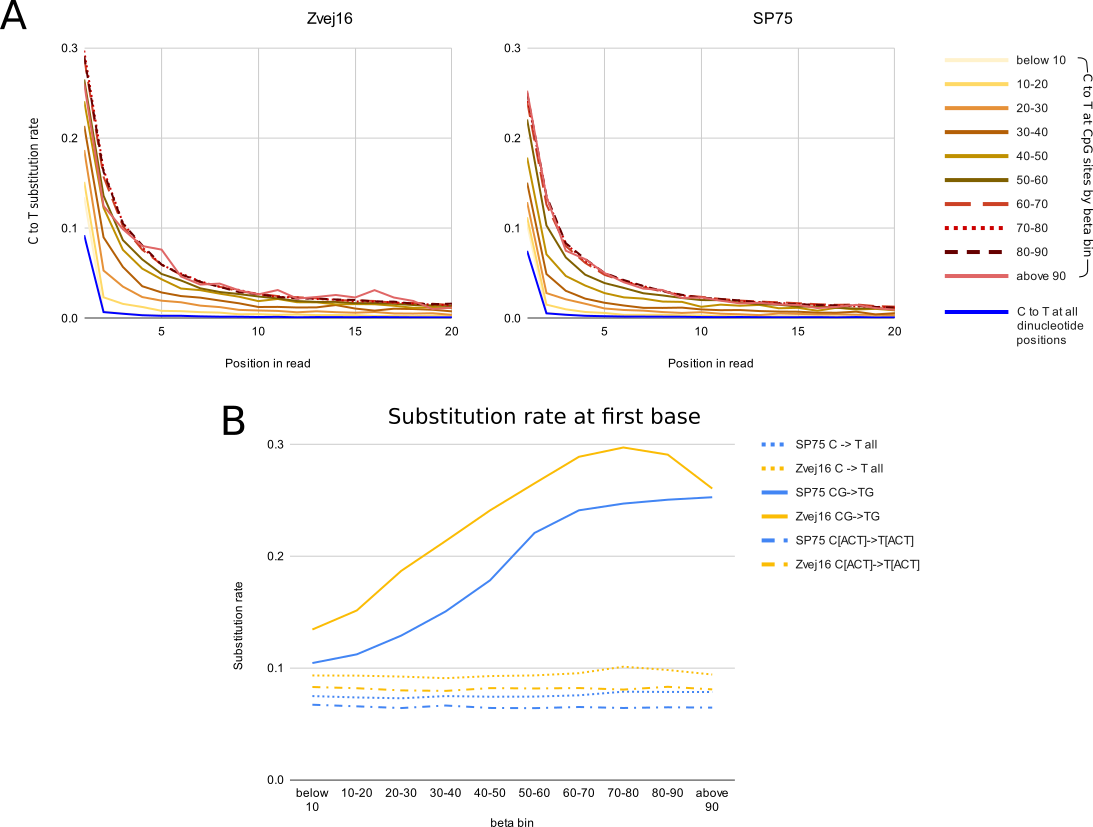


Figure S28. Deamination rate by methylation rate. A. The C to T substitution rate compared to the reference genome (C in the reference and T in the sequenced reads) of both SP75 and Zvej16 non-bisulfite treated high coverage data at the 5’ end. All but the blue line are the C to T rate only at CpG positions colored by methylation rate (beta bin). The blue line shows the C to T rate of all positions and all methylation rates. As the high coverage genomes are partially UDG treated, the first position still has a deamination signal. B. The C to T substitution rate by methylation rate (beta bin) at the first position of the read only. The C to T rate of all positions, as well as the C to T rate at CpG and non-CpG positions are shown.
