# Supplementary material for: Improved detection of methylation in ancient DNA
Source: Genome Biol. 2024 Oct 10;25:261. doi: 10.1186/s13059-024-03405-5 (PMC11465500; doi:10.1186/s13059-024-03405-5)
Supplement: Supplementary file 2 — Additional file 2: Review history. [file 13059_2024_3405_MOESM2_ESM.docx]

**1^st^ round**

**Reviewer 1**

Given the scarcity of literature on the fascinating topic of Paleoepigenomics I read the manuscript by Sawyer et al. entitled "Improved detection of methylation in ancient DNA" with great interest.
The manuscript did not disappoint and provides a very thorough assessment of laboratory methods enabling to detect "real" methylation patterns rather than computationally inferred ones. The study is a significant contribution to the field and will be read by many colleagues working in the field and being interested in epigenetic modifications in modern and ancient specimen to elucidate respective evolutionary changes.
Despite my enthusiasm for the study, I would like the authors to consider the points mentioned here.

My major concern is the suggested coverage (0.4) of the genomes the authors have applied a bisulfite treatment to and still recover methylation signals. While this is a great achievement and would now result in the inclusion of samples previously excluded from paleoepigenomic studies due to low coverage, I wonder what this means for a population-wide assessment of methylation landscapes. The first paleoepigenomic results were generated from single individuals and compared to a pool of modern data. However, the inferences drawn from such a study set-up are a bit limited in terms of investigating adaptive changes of entire populations or enlightening changes associated with various epochs in the history of humans. Hence my question would be: Given such a low coverage, how likely is it to recover methylation patterns in the same regions and thus evaluate hypo- or hypermethylation contexts of those? I would appreciate, if the authors could elaborate on this in the discussion, as this seems to me a crucial point in light of trends including more individuals in paleogenomic studies.

Another issue relates to the age of some of the additional samples being comparatively young (see Table 1). I would like to ask the authors to briefly discuss the effect of sample age on the potential of assessing methylation pattern given the various states of preservation. The authors appropriately discuss the effects of the many treatments on the DNA yield etc, but given that in ancient DNA research, almost every sample behaves differently it would be great to see a short discussion on this topic.


Minor comments:
-please include a sentence on methylation status of bases other than Cytosine in the introduction. Although these are not of relevance for most vertebrates, they still exist and are worth being mentioned.
-P12LL306: Please reformulate the sentence: "In strand 1, all treatments cause as few as 7 Cs to be deaminated, with the bisulfite treatment having the highest amount of Cs not deamintaed at 15.7%...". The authors change the focus from deaminated to non-deaminated, which to me is a bit confusing, and I would prefer to be consistent and either talk about deaminated or non-deaminated throughout the sentence.
-Just out of curiosity; at the end of the first paragraph at page 15 the authors talk about the effect of DNA concentration and the ability of detecting mCs in non-CpG context. Have the authors by any chance investigated a correlation of the read mapping-quality at the respective sites? This is just an idea but gets some relevance for low-coverage genomes as there are not many reads covering a specific site to start with and if there is a bias that would be worth mentioning.
-Throughout the method section (pages 23-24), whenever referring to volumes of extracts used in various steps, I would recommend starting the sentence with the required/necessary quantity of DNA and then provide the volumes needed to achieve this in brackets, similar to this: "the bisulfite treatment requires a minimum of XXX ng DNA (equivalent to 1-10ul DNA extract).
-Fig.1: In the EM-seq pipeline using ds libraries at the very bottom where the amplicons are depicted you can see one C in the priming site still being methylated (upper strand). I presume this should be a normal C like the other Cs in the primer region.
-Fig2B: Could the arrangement of the barplots be changed and start with the sslib only, like in the arrangement of 2A.

**Reviewer 2**

The authors of this manuscript have evaluated the efficacy of bisulfite and enzymatic methylation treatments on ancient DNA (aDNA), concluding that both methods preserve sufficient DNA for analysis. Particularly, bisulfite treatment, when combined with a single-stranded library preparation, exhibited minimal yield reduction and bias, enabling cost-effective methylation measurement even at low coverage. This broadens the scope for studying ancient samples. However, significant revisions are needed before publication. Some technical queries that readers may have or areas requiring further clarification have been outlined below.
Major comments:
1. My primary concern revolves around the postmortem damage to aDNA. How can we ensure the accuracy of the methods presented by the authors and how can we compare them with modern data?
2. Barouch et al. (2024) have proposed methods for reconstructing aDNA methylation maps. It is crucial to compare these methods to evaluate the robustness of the new approaches.
3. The results section should present data directly to delineate the advantages of the methods outlined in this paper for detecting ancient DNA methylation. Currently, there is excessive background information, making it challenging to discern the main findings immediately. For instance, lines 422-442.
4. In modern research, numerous methylation markers related to bone have been identified (Virginia, 2021). Have the authors compared the methylation map produced in this study with recent markers published?
Minor comments:
1. Lines 122-159: The text description is overly detailed and needs streamlining, especially the introduction.
2. Lines 148-194: The logical structure of the narrative is unclear. It could start with the challenges of detecting ancient DNA, followed by a description of the methods and recent research advances.
3. Lines 207-223: This section, detailing the samples and methods used for methylation testing, should be moved to the results.
4. Line 225: The title should better summarize the subsequent content.
5. Lines 227-272: A table should be included to describe and compare the 7 methods and their differences in methylation characterization.
6. Lines 289-312: This section should align with paragraphs 276-285.
7. Figures 2B and 3B: A significance level needs to be added.
8. Legends in Figures 2C and D: The order of the corresponding methods should be included after the current legend.

**Authors’ response**

Reviewer 1:

Given the scarcity of literature on the fascinating topic of Paleoepigenomics I read the manuscript by Sawyer et al. entitled "Improved detection of methylation in ancient DNA" with great interest.

The manuscript did not disappoint and provides a very thorough assessment of laboratory methods enabling to detect "real" methylation patterns rather than computationally inferred ones. The study is a significant contribution to the field and will be read by many colleagues working in the field and being interested in epigenetic modifications in modern and ancient specimen to elucidate respective evolutionary changes.

Despite my enthusiasm for the study, I would like the authors to consider the points mentioned here.

We thank the reviewer very much for the positive comments and address their points below.

Reviewer comment:

My major concern is the suggested coverage (0.4) of the genomes the authors have applied a bisulfite treatment to and still recover methylation signals. While this is a great achievement and would now result in the inclusion of samples previously excluded from paleoepigenomic studies due to low coverage, I wonder what this means for a population-wide assessment of methylation landscapes. The first paleoepigenomic results were generated from single individuals and compared to a pool of modern data. However, the inferences drawn from such a study set-up are a bit limited in terms of investigating adaptive changes of entire populations or enlightening changes associated with various epochs in the history of humans. Hence my question would be: Given such a low coverage, how likely is it to recover methylation patterns in the same regions and thus evaluate hypo- or hypermethylation contexts of those? I would appreciate, if the authors could elaborate on this in the discussion, as this seems to me a crucial point in light of trends including more individuals in paleogenomic studies.

Response:

Thank you for raising this point. We have expanded on this point in the discussion (lines 515 to 520).

Reviewer comment:

Another issue relates to the age of some of the additional samples being comparatively young (see Table 1). I would like to ask the authors to briefly discuss the effect of sample age on the potential of assessing methylation pattern given the various states of preservation. The authors appropriately discuss the effects of the many treatments on the DNA yield etc, but given that in ancient DNA research, almost every sample behaves differently it would be great to see a short discussion on this topic.

Response:

This is a good point, however after thousands of aDNA samples sequenced so far there is still not always a clear correlation between age and DNA preservation. DNA preservation can be linked to age, but it is also affected by temperature, temperature fluctuations, pH, humidity, etc. So a much older sample that was kept at a more stable temperature and better pH can easily have better DNA preservation than a much younger sample that has been exposed to high or fluctuating temperatures. As this is not clear in our manuscript, we have addressed this point (lines 345 to 348).

Minor comments:

Reviewer comment:

-please include a sentence on methylation status of bases other than Cytosine in the introduction. Although these are not of relevance for most vertebrates, they still exist and are worth being mentioned.

Response:

We have added a sentence and reference to the introduction to mention methylation of adenines.

Reviewer comment:

-P12LL306: Please reformulate the sentence: "In strand 1, all treatments cause as few as 7 Cs to be deaminated, with the bisulfite treatment having the highest amount of Cs not deamintaed at 15.7%...". The authors change the focus from deaminated to non-deaminated, which to me is a bit confusing, and I would prefer to be consistent and either talk about deaminated or non-deaminated throughout the sentence.

Response:

Thank you for pointing this out, we have changed the sentence to keep the focus on deaminated Cs.

Reviewer comment:

-Just out of curiosity; at the end of the first paragraph at page 15 the authors talk about the effect of DNA concentration and the ability of detecting mCs in non-CpG context. Have the authors by any chance investigated a correlation of the read mapping-quality at the respective sites? This is just an idea but gets some relevance for low-coverage genomes as there are not many reads covering a specific site to start with and if there is a bias that would be worth mentioning.

Response:

This is an interesting idea. However, we filter for mapping quality before performing any analyses. Our mapping quality cut-off is 30, which corresponds to a Phred-scaled probability of approximately 1 in 1,000 (or 0.001) that the alignment of a read is incorrect. If we included lower-quality mapped reads, it would make sense to check for biases caused by lower-quality mapping, however, we reduce that bias possibility by filtering for high-quality mapped reads to begin with.

Reviewer comment:

-Throughout the method section (pages 23-24), whenever referring to volumes of extracts used in various steps, I would recommend starting the sentence with the required/necessary quantity of DNA and then provide the volumes needed to achieve this in brackets, similar to this: "the bisulfite treatment requires a minimum of XXX ng DNA (equivalent to 1-10ul DNA extract).

Response:

We have added the ng of DNA extract used to each method description. As we did not test a large range of input ng of a single extract to test exactly how much input is needed, and as the minimum ng is also dependent on percent endogenous and that changes for each sample, we did not add a recommendation for the minimum required.

Reviewer comment:

-Fig.1: In the EM-seq pipeline using ds libraries at the very bottom where the amplicons are depicted you can see one C in the priming site still being methylated (upper strand). I presume this should be a normal C like the other Cs in the primer region.

Response:

Thank you for pointing this out. We have fixed it.

Reviewer comment:

-Fig2B: Could the arrangement of the barplots be changed and start with the sslib only, like in the arrangement of 2A.

Response:

We have changed the order as suggested.

Reviewer 2:

The authors of this manuscript have evaluated the efficacy of bisulfite and enzymatic methylation treatments on ancient DNA (aDNA), concluding that both methods preserve sufficient DNA for analysis. Particularly, bisulfite treatment, when combined with a single-stranded library preparation, exhibited minimal yield reduction and bias, enabling cost-effective methylation measurement even at low coverage. This broadens the scope for studying ancient samples. However, significant revisions are needed before publication. Some technical queries that readers may have or areas requiring further clarification have been outlined below.

Many thanks for the review, we have addressed each point below.

Major comments:

Reviewer comment:

My primary concern revolves around the postmortem damage to aDNA. How can we ensure the accuracy of the methods presented by the authors and how can we compare them with modern data?

Response:

Thank you for addressing this concern. We have delved deeper into this issue by producing additional analyses: please see Figure S28, Table S9, Table S10 and the results and discussion section for our analyses.

Reviewer comment:

Barouch et al. (2024) have proposed methods for reconstructing aDNA methylation maps. It is crucial to compare these methods to evaluate the robustness of the new approaches.

Response:

The Barouch et al. method is an important advance merging lower coverage published non-bisulfite-treated data to reconstruct methylation maps. To truly compare the two methods, we would need multiple samples with a combined coverage greater than 12-fold and with the same genetic ancestry components. This latter point is very important as differing genetic ancestry can bias methylation signals (see Fagny et al 2015). We would also need the data for the published genomes to come from the same laboratory (see Çokoğlu et al 2023 -- BioRxiv). We only have higher coverage for two of our samples, but even the captured libraries would not have 12-fold coverage for the two combined. Even if we did produce this coverage though, one is a mesolithic hunter-gatherer while the other is a neolithic farmer, so combining these two would not be prudent due to their different genetic ancestry. As it is an important paper in the field, we have mentioned this paper in our manuscript (line 207).

Reviewer comment:

The results section should present data directly to delineate the advantages of the methods outlined in this paper for detecting ancient DNA methylation. Currently, there is excessive background information, making it challenging to discern the main findings immediately. For instance, lines 422-442.

Response:

Thank you for pointing this out. We have rewritten the highlighted section to make the results clearer with less background.

Reviewer comment:

In modern research, numerous methylation markers related to bone have been identified (Virginia, 2021). Have the authors compared the methylation map produced in this study with recent markers published?

Response:

There is, unfortunately, a dearth of modern methylation studies using bones. The “Visconti et al. DNA Methylation Signatures of Bone Metabolism in Osteoporosis and Osteoarthritis Aging-Related Diseases: An Updated Review. Int J Mol Sci. 2021 Apr 19;22(8):4244” paper is a great review of the various studies that have used blood, cartilage and bone cells to study osteoporosis and osteoarthritis, almost exclusively in post-menopausal women. We have two male samples, of which one is a 2-3-year-old child, neither of which have any indicators of bone disease (see updated supplemental materials/supplemental information for SP75 and Mathieson 2018 for Zvej16). In addition, both samples were taken from petrous bone, which is less associated with osteoporosis that has a unique development path compared to other bones.

Instead, we would need a large sample size to study bone diseases in ancient samples, ideally from hip, wrist or spinal bones, with the appropriate paleopathological determinations of osteoporosis/osteoarthritis to properly study these markers. Indeed it is not common to find clear indications of osteoporosis in skeletal material. As we only have two samples, we would not be able to make any biological inferences, as our focus is to describe a method to be used for future biological questions. We have included this study direction as a future application made possible with our method in the discussion.

Minor comments:

Reviewer comment:

Lines 122-159: The text description is overly detailed and needs streamlining, especially the introduction.

Response:

We have made the text shorter to remove extra information.

Reviewer comment:

Lines 148-194: The logical structure of the narrative is unclear. It could start with the challenges of detecting ancient DNA, followed by a description of the methods and recent research advances.

Response:

We have rewritten this section to more clearly follow a narrative.

Reviewer comment:

Lines 207-223: This section, detailing the samples and methods used for methylation testing, should be moved to the results.

Response:

Thank you, in the revised version this section is in the results section.

Reviewer comment:

Line 225: The title should better summarize the subsequent content.

Response:

We have rephrased the title.

Reviewer comment:

Lines 227-272: A table should be included to describe and compare the 7 methods and their differences in methylation characterization.

Response:

We have made it clearer that this section is supported by Figure 1. Since the different methods and treatments are not linear, a table could be more confusing than the current graphical explanation in Figure 1.

Reviewer comment:

Lines 289-312: This section should align with paragraphs 276-285.

Response:

We have combined these sections to make the positive control types clearer.

Reviewer comment:

Figures 2B and 3B: A significance level needs to be added.

Response:

We have added significance levels to the figures.

Reviewer comment:

Legends in Figures 2C and D: The order of the corresponding methods should be included after the current legend.

Response:

We have added the order of methods in the legend.

**2^nd^ round**

**Reviewer 1**

I would like to thank the authors for addressing my concerns and incorporating adjustments according to my comments. I don't have any further issues.

**Reviewer 2**

Thank you for the detailed response and significant revisions made to the manuscript. The article has indeed addressed many of my previous concerns regarding the methods for methylation detection in ancient DNA (aDNA). However, I still have some reservations, particularly about the accuracy of the results. Here are my specific comments and suggestions:
Accuracy Verification: The main focus of this study is the accuracy of the methylation detection methods in aDNA. To validate this, I suggest two ways, first the authors should identify existing publicly available ancient samples with methylation maps and perform a correlation comparison. This would help assess the reliability of the new methods in practical applications. Though I previously mentioned two reference papers whose data the authors might not wish to use, they should still seek other publicly available methylation data for comparison. This would enhance the credibility and overall validity of the results. No matter the genetic background, age and other biological matters, the methylation values are conserved in specific tissues. Second, prediction existing conclusions, as mentioned in the abstract mentions, “Reconstructing premortem DNA methylation levels in ancient DNA (aDNA) has led to breakthrough studies such as the prediction of anatomical features of the Denisovan.” I would like the authors to further discuss whether the sequencing data can validate the existing predictions. If not, please explain the reasons and discuss the directions for improvement.
Overall, the authors have made substantial efforts in improving the methods, which is commendable. However, to enhance the scientific rigor and reliability of the findings, I recommend that the authors further conduct data comparisons and validations. This would not only support the effectiveness of the new methods but also provide a more solid foundation for future aDNA methylation research.
I hope these suggestions are helpful to the authors and the journal.

**Further advice from Reviewer 1**

I now had the time to check this issue in more detail and have to say that I agree with the authors and with you.

I do understand the motivation of reviewer 2, but I feel his proposal goes beyond the scope of the study by Sawyer et al. They demonstrate a new method that can be applied in the lab to reconstruct methylation maps and provide ample evidence that the performance of their method is comparable to previously applied approaches.

In order to compare the outcome of the approach to other existing methylation maps, they would need to process the exact same samples with all approaches under consideration….which seems quite elaborate and in part they did. Just comparing the results from one paper to another does not really help here as methylation maps are very tricky to establish and also variable within populations/tissues etc (in particular for ancient specimens).

To be honest, I do not see a way how Sawyer et al. would be able to sufficiently address this issue in a timely manner.

To sum it up: I think that at this stage of the process, it will be difficult to address the point of the reviewer as it requires the inclusion of new, elaborate lab work.
